# Supplementary material for: HMFGraph: Novel Bayesian approach for recovering biological networks
Source: PLoS Comput Biol. 2025 Oct 30;21(10):e1013614. doi: 10.1371/journal.pcbi.1013614 (PMC12591435; doi:10.1371/journal.pcbi.1013614)
Supplement: S1 Text — Includes all supporting information materials. (PDF) [file pcbi.1013614.s001.pdf]

HMFGraph: Novel Bayesian approach for recovering biological networks, Supporting information

Aapo E. Korhonen, Olli Sarala, Tuomas Hautamäki, Markku Kuismin, and Mikko J. Sillanpää

October 21, 2025

Contents

A Full conditional distributions **2**

B Gibbs sampler **5**

C GEM algorithm **6**

D Hyperparameter selection **7**

E Binary search for α value **8**

F Variance-Gamma distribution's relation to off-diagonal elements **19**

G The optimal credible interval **19**

H Measuring model accuracy **19**

I Technical details on reproducing the results **21**

J Comparisons of network recovery **23**

K Riboflavin dataset **28**

L American gut data **30**

M Proof of proper posterior distribution: improper prior **33**

N Proof of proper posterior distribution: gamma prior **35**

A Full conditional distributions

The likelihood function of the Bayesian Gaussian graphical models is

$$Y_j|\Omega \sim N(0, \Omega^{-1}), \text{for all }, \; j = 1, \ldots, n,$$

$$p(Y|\Omega) = (2\pi)^{-\frac{np}{2}} |\Omega|^{\frac{n}{2}} \exp\left[-\frac{1}{2}\sum_{j=1}^{n}(y_j - \mu)^T \Omega (y_j - \mu)\right]$$

$$\propto |\Omega|^{\frac{n}{2}} \exp\left[-\frac{1}{2}\text{tr}(nS\Omega)\right]. \tag{A}$$

The priors for parameters Ω and Φ are Wishart distributions:

$$\Omega|\Phi \sim W(\nu, ((\nu - p - 1)\Phi)^{-1}),$$

$$p(\Omega|\Phi) = \frac{|\Omega|^{\frac{\nu-p-1}{2}}}{2^{\frac{\nu p}{2}} |((\nu - p - 1)\Phi)^{-1}|^{\frac{\nu}{2}} \Gamma_p(\frac{\nu}{2})} \exp\left[-\frac{1}{2}\text{tr}((\nu - p - 1)\Phi\Omega)\right], \tag{B}$$

$$\Phi|B \sim W(\delta + p - 1, (\delta + p - 1)B^{-1}),$$

$$p(\Phi|B) = \frac{|\Phi|^{\frac{\delta+p-1-p-1}{2}}}{2^{\frac{(\delta+p-1)p}{2}} |((\delta + p - 1)B)^{-1}|^{\frac{\delta+p-1}{2}} \Gamma_p(\frac{(\delta+p-1)}{2})} \exp\left[-\frac{1}{2}\text{tr}((\delta + p - 1)B\Phi)\right]. \tag{C}$$

Above, B is a diagonal matrix ($b_{ij} = 0, i \neq j$). The prior for it is a gamma distribution with scale-rate parameterization:

$$b_{ii} \sim \text{Gamma}(\text{shape} = \epsilon_1, \text{rate} = \epsilon_2), \tag{D}$$

$$p(b_{ii}) = \frac{\epsilon_2^{\epsilon_1}}{\Gamma(\epsilon_1)} b_{ii}^{\epsilon_1 - 1} \exp\left[-\epsilon_2 b_{ii}\right]. \tag{E}$$

The gamma prior can be replaced with a scale-invariant flat prior on a logarithmic scale, which is an improper prior. It is

$$p(b_{ii}) = \frac{1}{b_{ii}}, \; b_{ii} \in]0, +\infty[. \tag{F}$$

The full conditional posterior density for parameter Ω is

$$\begin{aligned}
p(\Omega|\cdot) &\propto |\Omega|^{\frac{n}{2}} \exp\left[-\frac{1}{2}\text{tr}(nS\Omega)\right] \exp\left[-\frac{1}{2}\text{tr}((\nu-p-1)\Phi\Omega)\right] |\Omega|^{\frac{\nu-p-1}{2}} \\
&= |\Omega|^{\frac{\nu+n-p-1}{2}} \exp\left[-\frac{1}{2}\text{tr}(nS\Omega)\right] \exp\left[-\frac{1}{2}\text{tr}((\nu-p-1)\Phi\Omega)\right] \\
&= |\Omega|^{\frac{\nu+n-p-1}{2}} \exp\left[-\frac{1}{2}\text{tr}((nS+(\nu-p-1)\Phi)\Omega)\right] \\
\Omega|\cdot &\sim W(\nu+n, (nS+(\nu-p-1)\Phi)^{-1}). \qquad \text{(G)}
\end{aligned}$$

The full conditional posterior density for parameter Φ is

$$\begin{aligned}
p(\Phi|\cdot) &\propto |\Phi|^{\frac{\delta+p-1-p-1}{2}} \exp\left[-\frac{1}{2}\text{tr}((\delta+p-1)B\Phi)\right] |\Phi|^{\frac{\nu}{2}} \exp\left[-\frac{1}{2}\text{tr}((\nu-p-1)\Phi\Omega)\right] \\
&= |\Phi|^{\frac{\delta+\nu+p-1-p-1}{2}} \exp\left[-\frac{1}{2}\text{tr}((\delta+p-1)B\Phi) - \frac{1}{2}\text{tr}((\nu-p-1)\Phi\Omega)\right] \\
&= |\Phi|^{\frac{\delta+\nu+p-1-p-1}{2}} \exp\left[-\frac{1}{2}(\text{tr}((\delta+p-1)B\Phi) + \text{tr}((\nu-p-1)\Omega\Phi)\right] \\
&= |\Phi|^{\frac{\delta+\nu+p-1-p-1}{2}} \exp\left[-\frac{1}{2}(\text{tr}((\delta+p-1)B\Phi + (\nu-p-1)\Omega\Phi)\right] \\
&= |\Phi|^{\frac{\delta+\nu+p-1-p-1}{2}} \exp\left[-\frac{1}{2}(\text{tr}((((\delta+p-1)B + (\nu-p-1)\Omega)\Phi)\right], \\
\Phi|\cdot &\sim W(\delta+\nu+p-1, ((\delta+p-1)B+(\nu-p-1)\Omega)^{-1}). \qquad \text{(H)}
\end{aligned}$$

The full conditional posterior density for each b_{ii} is

$$\begin{aligned}
p(b_{ii}|\cdot) &\propto b_{ii}^{-\epsilon_1-1}|(\delta+p-1)B|^{-\frac{\delta+p-1}{2}} \exp\left[-\frac{1}{2}\text{tr}((\delta+p-1)B\Phi\right] \exp\left[-\epsilon_2 b_{ii}\right] \\
&\propto b_{ii}^{-\epsilon_1-1} b_{ii}^{-\frac{\delta+p-1}{2}} \exp\left[-b_{ii}(\frac{1}{2}(\delta+p-1)\phi_{ii}+\epsilon_2)\right] \\
&\propto b_{ii}^{-(\frac{\delta+p-1}{2}+\epsilon_1)-1} \exp\left[-b_{ii}(\frac{1}{2}(\delta+p-1)\phi_{ii}+\epsilon_2)\right], \\
b_{ii}|\cdot &\sim \text{Gamma}(\text{shape} = (\delta+p-1)/2+\epsilon_1, \text{rate} = (\delta+p-1)\phi_{ii}/2+\epsilon_2). \qquad \text{(I)}
\end{aligned}$$

The full conditional posterior density for each b_{ii}, if the improper prior (F) is

used, is

$$
\begin{aligned}
p(b_{ii}|\cdot) &\propto b_{ii}^{-1}|(\delta+p-1)B|^{\frac{\delta+p-1}{2}} \exp\left[-\frac{1}{2}\mathrm{tr}((\delta+p-1)B\Phi)\right] \\
&\propto b_{ii}^{-1} b_{ii}^{\frac{\delta+p-1}{2}} \exp\left[-\frac{1}{2}(\delta+p-1)b_{ii}\phi_{ii}\right] \\
&\propto b_{ii}^{\frac{\delta+p-1}{2}-1} \exp\left[-\frac{1}{2}b_{ii}(\delta+p-1)\phi_{ii}\right], \\
b_{ii}|\cdot &\sim \mathrm{Gamma}(\mathrm{shape}=(\delta+p-1)/2, \mathrm{rate}=(\delta+p-1)\phi_{ii}/2). \qquad \text{(J)}
\end{aligned}
$$

As seen in the previous equation, both priors for the diagonal of B produce a similar full conditional distribution. The difference can be seen in the shape and rate parameters. We will use the gamma prior (D) for all results. If the improper prior is used (F), the full conditional is the same as using Equation (I), but with $\epsilon_1 = \epsilon_2 = 0$.

Additionally, all modes of these distributions are analytically available. The conditional maximum for (G) is the same as the mode of the full conditional posterior distribution: $\mathrm{argmax}_\Omega(\mathrm{P}(\Omega|\cdot)) = \mathrm{Mode}(\Omega|\cdot)$, and the same is true for the parameters Φ and B. The modes for (G) and (H) are

$$
\begin{aligned}
\mathrm{Mode}(\Omega|\cdot) &= (\nu+n-p-1)((\nu-p-1)\Phi+nS)^{-1}, \\
\mathrm{Mode}(\Phi|\cdot) &= (\delta+\nu-2)((\delta+p-1)B+(\nu-p-1)\Omega)^{-1}.
\end{aligned}
$$

Because of our scaling modification, we can replace ν and δ with new parameters α and β respectively:

$$
\begin{aligned}
\alpha &= \frac{\nu-p-1}{\nu+n-p-1}, \\
\beta &= \frac{\delta+p-1}{\delta+\nu-2}.
\end{aligned}
$$

Modes are then

$$
\begin{aligned}
\mathrm{Mode}(\Omega|\cdot) &= (\alpha\Phi+(1-\alpha)S)^{-1}, \qquad \text{(K)} \\
\mathrm{Mode}(\Phi|\cdot) &= (\beta B+(1-\beta)\Omega)^{-1}. \qquad \text{(L)}
\end{aligned}
$$

The mode for each b_{ii} is

$$
\mathrm{Mode}(b_{ii}|\cdot) = \frac{\mathrm{shape}-1}{\mathrm{rate}} = \frac{(\delta+p-1)/2+\epsilon_1-1}{(\delta+p-1)\phi_{ii}/2+\epsilon_2}. \qquad \text{(M)}
$$

For notational simplicity, we will still use δ in Equation (M). If the improper prior is used, the mode of the conditional distribution is the same as (M), but with $\epsilon_1 = \epsilon_2 = 0$.

Note that the mode of the Wishart distribution exists (or is defined) only if the degree of freedom is larger than $p+1$. Thus, the lower limit for δ is 1, and for ν it is $p+1$. The lower limit for α is then

$$\alpha = \frac{\nu - p - 1}{\nu + n - p - 1} = \frac{p + 1 - p - 1}{p + 1 + n - p - 1} = 0,$$

and the lower limit of β is

$$\beta = \frac{\delta + p - 1}{\delta + \nu - 2} = \frac{1 + p - 1}{1 + \nu - 2} = \frac{p}{\nu - 1}.$$

In our R implementation, we still use parameters ν and δ. Parameters α and β can be transformed to ν and δ with the following equations:

$$\nu = \frac{\alpha n + (1-\alpha)p + (1-\alpha)}{1-\alpha}, \tag{N}$$

$$\delta = \frac{\beta\nu - p + 1 - 2\beta}{1-\beta}. \tag{O}$$

The variance of the full conditional posterior distribution for off-diagonal elements of the precision matrix is

$$\mathrm{Var}(\omega_{ij}|\cdot) = (\nu + n)(w_{ij}^2 + w_{ii}w_{jj}), \tag{P}$$

where $W = [w_{ij}] = (nS + (\nu - p - 1)\Phi)^{-1}$ and w_{ij} its (ij) element. This can be used to estimate the variance of the posterior distribution:

$$\widehat{\mathrm{Var}}(\hat{\omega}_{ij}) = (\nu + n)(\hat{w}_{ij}^2 + \hat{w}_{ii}\hat{w}_{jj}), \tag{Q}$$

where $\widehat{W} = [\hat{w}_{ij}] = (nS + (\nu - p - 1)\widehat{\Phi})^{-1}$ and $\widehat{\Phi}$ is a MAP estimate of the parameter Φ. The variance (Q) is used to estimate the variance of the posterior distribution of off-diagonal elements of the precision matrix.

B Gibbs sampler

Here we introduce the Gibbs sampler for our model. Each parameter is sampled from its respective full conditional distribution during the sampling procedure. The Gibbs

sampler is simple to implement because we know all the full conditional distributions for each parameter.

The Gibbs sampler algorithm is

- Initialize parameters.
- Iterate K-number of times.
  - Sample $b_{ii}^{(k+1)}|\cdot \sim$ Gamma(shape $= (\delta + p - 1)/2 + \epsilon_1$, rate$=((\delta + p - 1)\phi_{ii}^{(k)})/2 + \epsilon_2)$.
  - Sample $\Phi^{(k+1)}|\cdot \sim W(\delta+\nu+p-1, ((\delta+p-1)B^{(k+1)}+(\nu-p-1)\Omega^{(k)})^{-1})$.
  - Sample $\Omega^{(k+1)}|\cdot \sim W(\nu + n, ((\nu - p - 1)\Phi^{(k+1)} + nS)^{-1})$.

C GEM algorithm

The E-step is an identity operation with our model:

$$Q(\Omega, \Phi, B|\Omega^{(k)}, \Phi^{(k)}, B^{(k)}) \equiv p(\Omega, \Phi, B|Y) \propto p(Y|\Omega)p(\Omega|\Phi)p(\Phi|B)p(B).$$

The conditional maximization steps:

Updating diagonal elements of B. For all $i \in 1 : p$:

$$\begin{aligned} b_{ii}^{(k+1)} &= \text{argmax}_{b_{ii}}(Q(\Omega^{(k)}, \Phi^{(k)}, B)) \\ &= \text{argmax}_{b_{ii}}(p(b_{ii}|\cdot)) = \frac{(\delta + p - 1)/2 + \epsilon_1 - 1}{(\delta + p - 1)\phi_{ii}^{(k)}/2 + \epsilon_2}. \end{aligned}$$

If the flat prior on a logarithmic scale is used for the parameter B, then $\epsilon_1 = \epsilon_2 = 0$.

Updating Φ:

$$\begin{aligned} \Phi^{(k+1)} &= \text{argmax}_{\Phi}(Q(\Omega^{(k)}, \Phi, B^{(k+1)})) \\ &= \text{argmax}_{\Phi}(p(\Phi|\cdot)) = (\beta B^{(k+1)} + (1 - \beta)\Omega^{(k)})^{-1}. \end{aligned}$$

Updating Ω:

$$\Omega^{(k+1)} = \text{argmax}_\Omega(Q(\Omega, \Phi^{(k+1)}, B^{(k+1)}))$$
$$= \text{argmax}_\Omega(p(\Omega|\cdot)) = (\alpha\Phi^{(k+1)} + (1-\alpha)S)^{-1}.$$

All full conditional distributions $p(\Phi|\cdot)$, $p(b_{ii}|\cdot)$ and $p(\Omega|\cdot)$ are known (G, I, H) and they have closed-form modes (or argmax) (see Equations K, L and M).

The proposed GEM algorithm will produce the MAP estimates of the parameters of the joint posterior distribution:

$$(\widehat{\Omega}, \widehat{\Phi}, \widehat{B}) = \text{argmax}_{\Omega,\Phi,B} p(\Omega, \Phi, B|Y).$$

D Hyperparameter selection

We found out by experiment that the rule-of-thumb equations $\alpha = 10 \cdot p/(10 \cdot p + n)$ and $\alpha = 2 \cdot p/(2 \cdot p + n)$ work well with datasets generated with the R packages `huge`, and `BDgraph`, respectively. This is illustrated in Fig C.

One possible way of selecting a suitable α value would be using the same empirical Bayes method that Leday and Richardson (2019) used with Fast BGGM. Their model is simpler than ours, using only an inverse-Wishart prior for the covariance matrix. A major benefit is the ability to analytically solve the marginal likelihood, from which a log marginal likelihood maximum for the α parameter is possible to attain. In equation form, it is

$$\hat{\nu} = \text{argmax}_\nu \left(\log \left(\frac{\pi^{-pn/2}\Gamma_p(\frac{\nu+n}{2})|(\nu-p-1)\Phi|^{\frac{\nu}{2}}}{\Gamma_p(\frac{\nu}{2})|(\nu-p-1)\Phi + nS|^{\frac{\nu+n}{2}}} \right) \right), \tag{R}$$

where $\hat{\nu}$ can be transformed to α with Equation (N). We propose that this equation could be used with our model to select an approximative value for α. For this approximation, the matrix Φ will be set to an identity matrix $\Phi = I_p$, which is not ideal.

Because the Equation (K) $(\alpha\Phi + (1-\alpha)S)^{-1}$ is similar to a linear shrinkage of the covariance matrix, or Ledoit-Wolf shrinkage (Ledoit and Wolf, 2004a,b), we could borrow some ideas from it on how an optimal shrinkage is selected. The Ledoit-Wolf estimator, or LW-estimator, for a covariance matrix is

$$\widehat{\Sigma}_{LW} = (1-\gamma) \cdot S + \gamma F,$$

where S is a sample covariance, F is a target matrix and γ is a shrinkage or penalization parameter. An optimal value for γ is fairly easy to calculate (Ledoit and Wolf, 2004a,b). Often, the target matrix F is selected to be a diagonal matrix, which raises the same problem as in Equation (R), namely the fact that the corresponding parameter Φ is not necessarily a diagonal matrix. Therefore, it would not be wise to use the same optimal shrinkage parameter γ value based on the LW-estimator's optimal shrinkage. However, the optimal value for LW-shrinkage γ could give insight for selecting a suitable α value.

E Binary search for α value

Parallelization cannot be utilized for the binary search, but in order to improve computation times, we use the so-called "warm start" trick (Li et al., 2015; Kim et al., 2018). This means that for the next GEM algorithm run with α_{new}, the previous results (with α_{old}) $(\widehat{\Omega}_{\alpha_{new}}, \widehat{\Phi}, \widehat{B})$ are used as its initial values. If α_{old} and α_{new} are close, the improvement for the convergence is noticeable. The binary search is presented in Algorithm A.

For α_{lower}, a value $p/(n+p)$ was used for all results presented in this manuscript. The α_{upper} was always set to 0.95.

Algorithm A Binary search for α value (*condition number constraint method*)

Require: max-iters, ϵ, p, n, $data$

$\Omega_{init} \leftarrow I_p$, $\Phi_{init} \leftarrow I_p$, $B_{init} \leftarrow I_p$ ▷ Initial values
$\alpha_{new} \leftarrow (\alpha_{lower} + \alpha_{upper})/2$
$\alpha \leftarrow \alpha_{upper}$
$\widehat{\Sigma}_{\text{LW}} \leftarrow$ Solve LW-estimate($data$)
for $i \leftarrow 1$ to max-iters **do**
  $(\widehat{\Omega}_{\alpha_{new}}, \widehat{\Phi}, \widehat{B}) \leftarrow$ GEM algorithm for HMFGraph$(data, \alpha_{new}, \Omega_{init}, \Phi_{init}, B_{init})$
  diff $\leftarrow$ (Cond($\widehat{\Sigma}_{\text{LW}}$) $-$ Cond($\widehat{\Omega}_{\alpha_{new}}$))/(Cond($\widehat{\Sigma}_{\text{LW}}$))
  if $0 <$ diff $< \epsilon$ **then**
    $\alpha \leftarrow \alpha_{new}$
    break
  else if diff < 0 **then**
    $\alpha_{lower} \leftarrow \alpha_{new}$
  else
    $\alpha_{upper} \leftarrow \alpha_{new}$
  end if
  $\alpha_{new} \leftarrow (\alpha_{lower} + \alpha_{upper})/2$
  $\Omega_{init} \leftarrow \widehat{\Omega}_{\alpha_{new}}$, $\Phi_{init} \leftarrow \widehat{\Phi}$, $B_{init} \leftarrow \widehat{B}$ ▷ Warm start trick
end for
return (α)

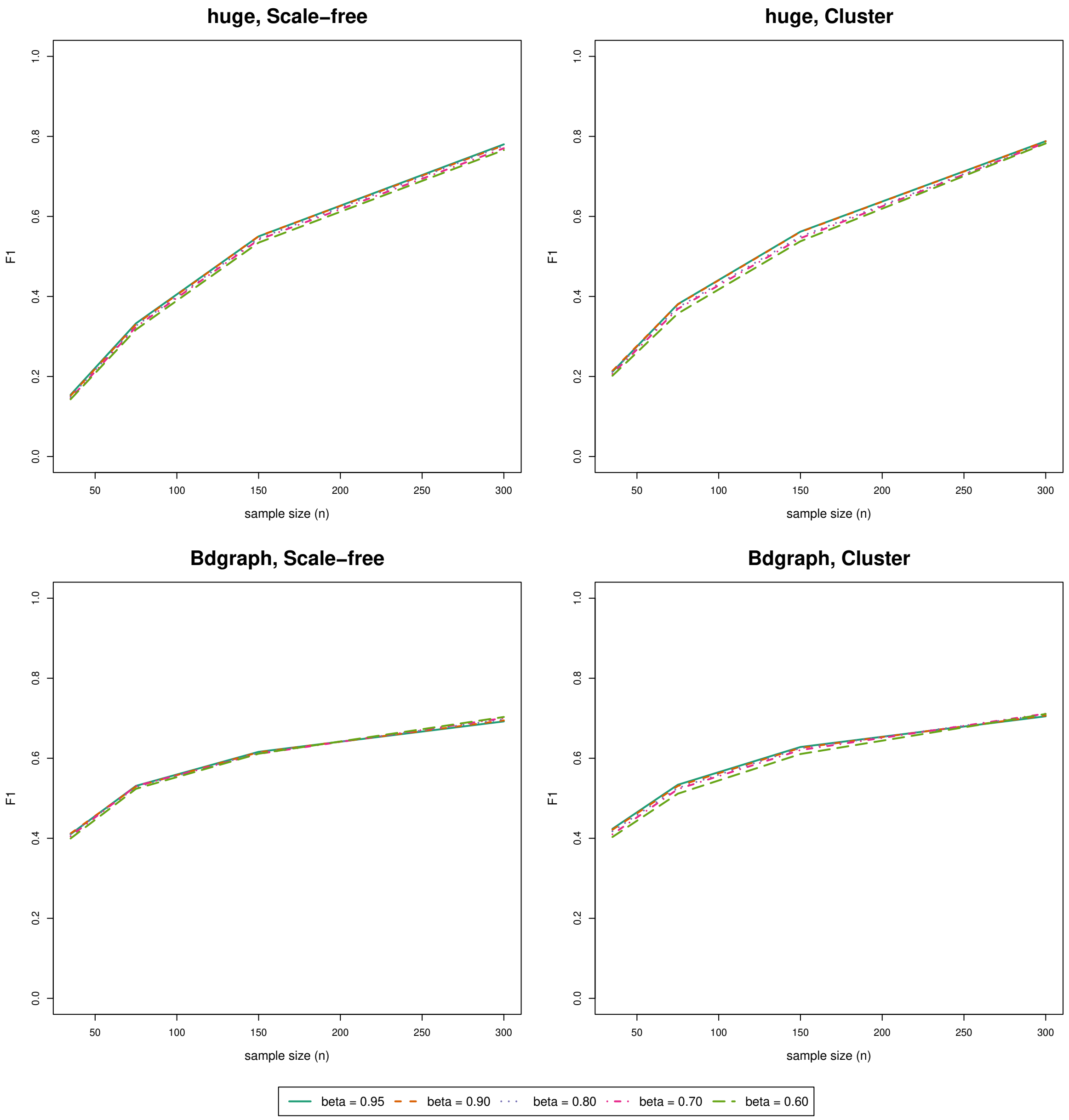

Fig A: Averages of F_1 values with different β values. For all β values, α was selected using the CC-method. HMFGraph with the optimal CI selected by maximizing the estimated F_1.

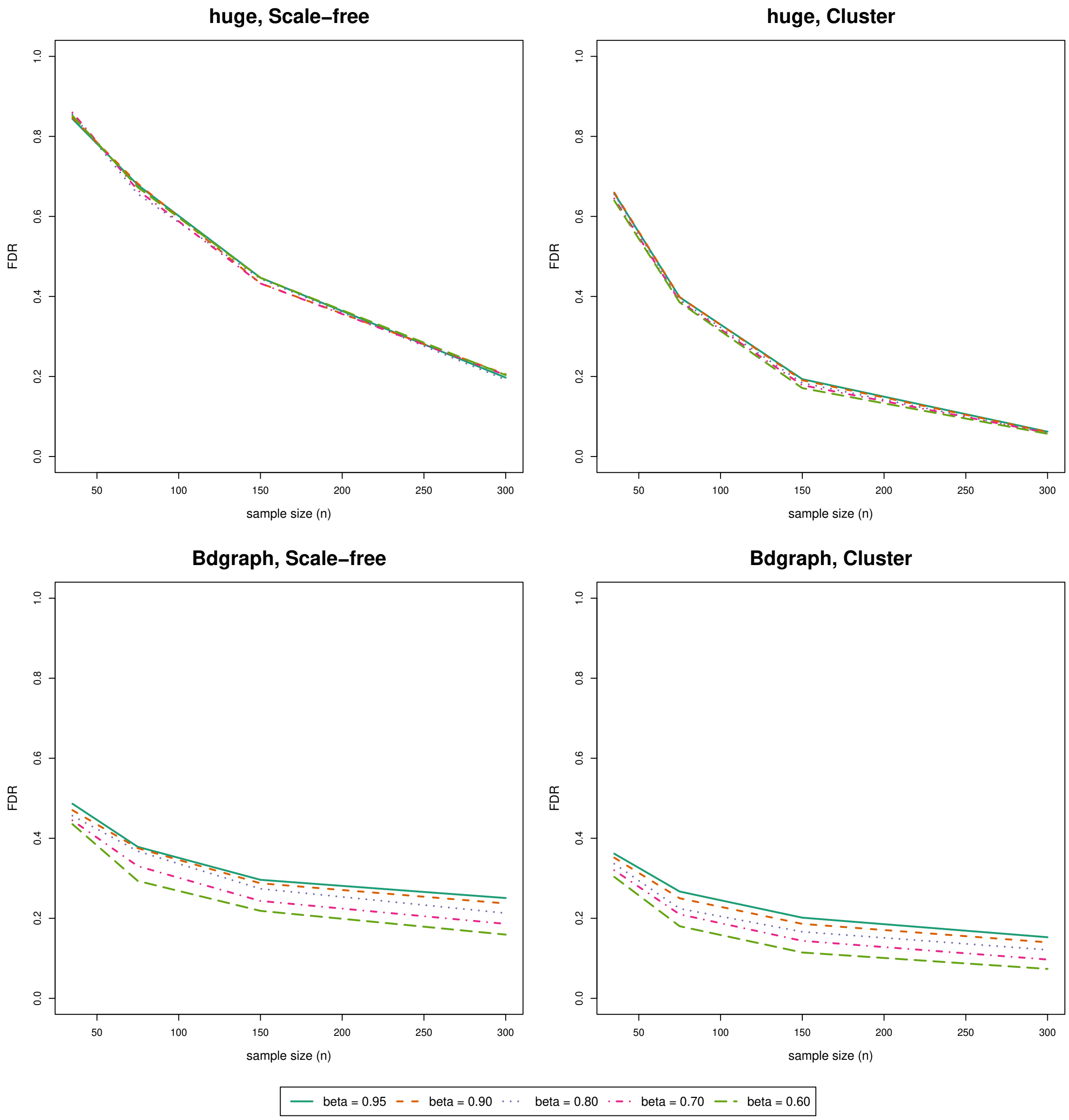

Fig B: Averages of FDR values with different β values. For all β values, α was selected using the CC-method. HMFGraph with the optimal CI selected by maximizing the estimated F_1.

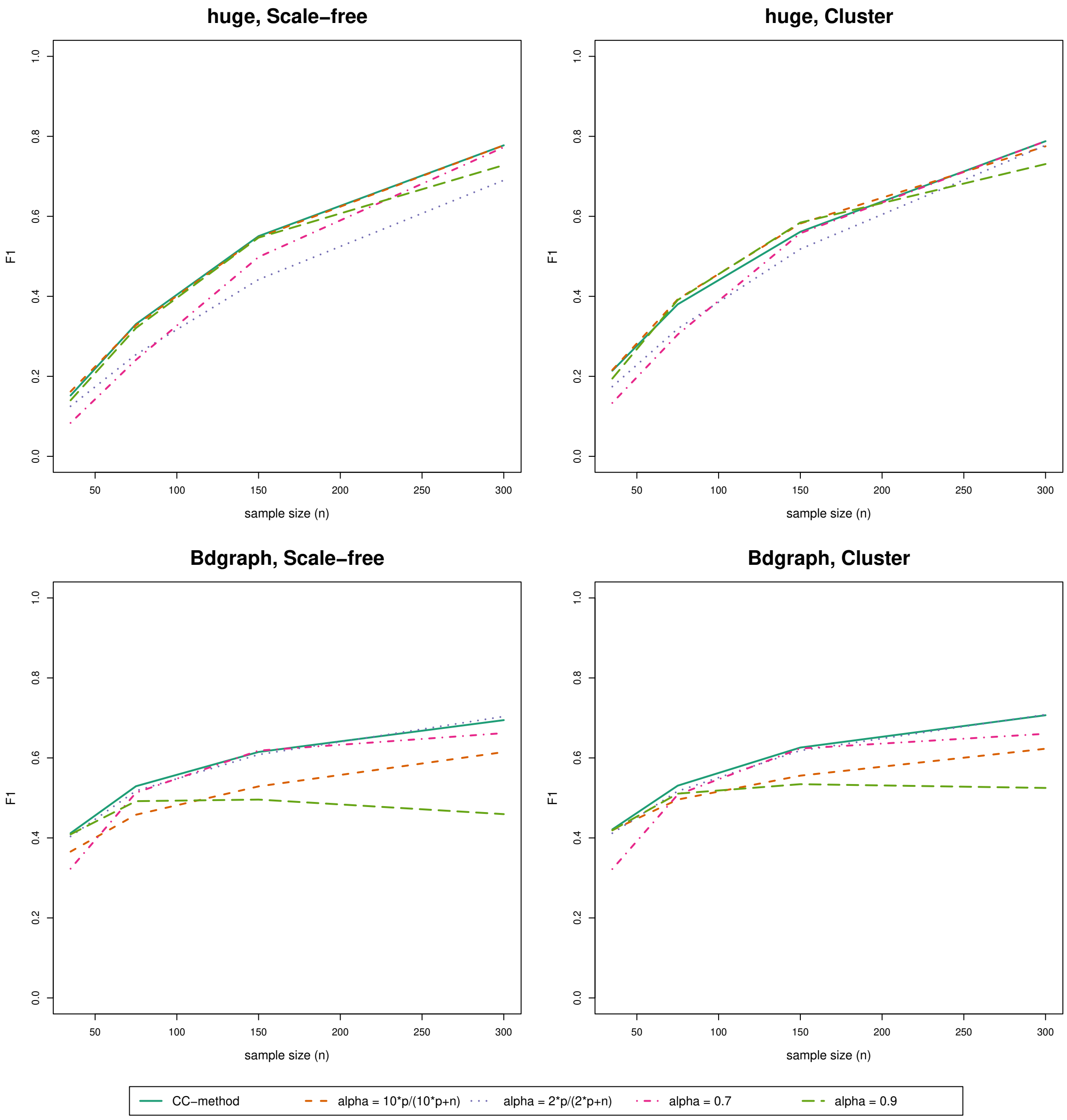

Fig C: Averages of F_1 values with different α values. For all α values, the parameter $\beta = 0.9$. HMFGraph with the optimal CI selected by maximizing the estimated F_1.

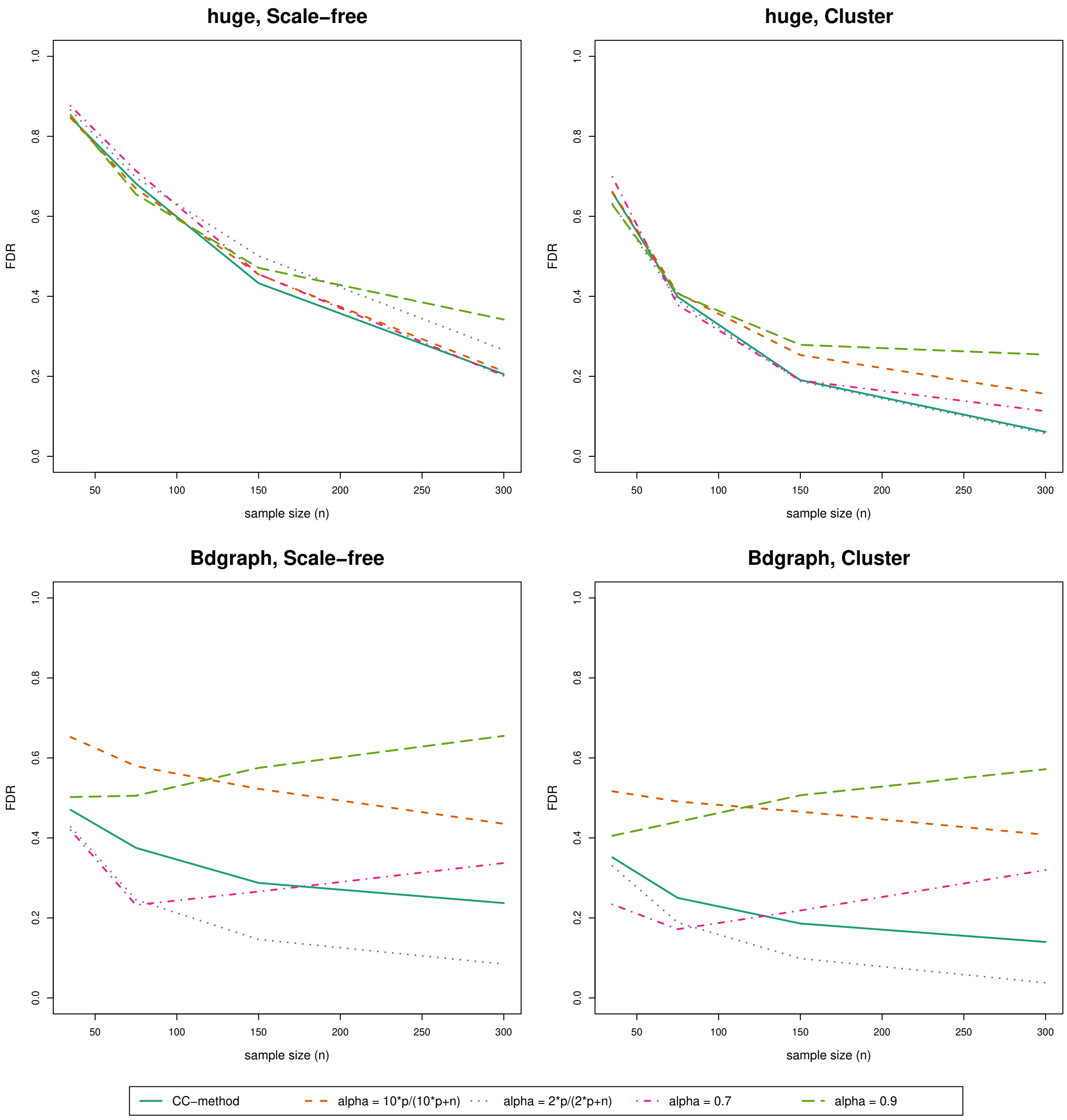

Fig D: Averages of FDR values with different α values. For all α values, the parameter $\beta = 0.9$. HMFGraph with the optimal CI selected by maximizing the estimated F_1.

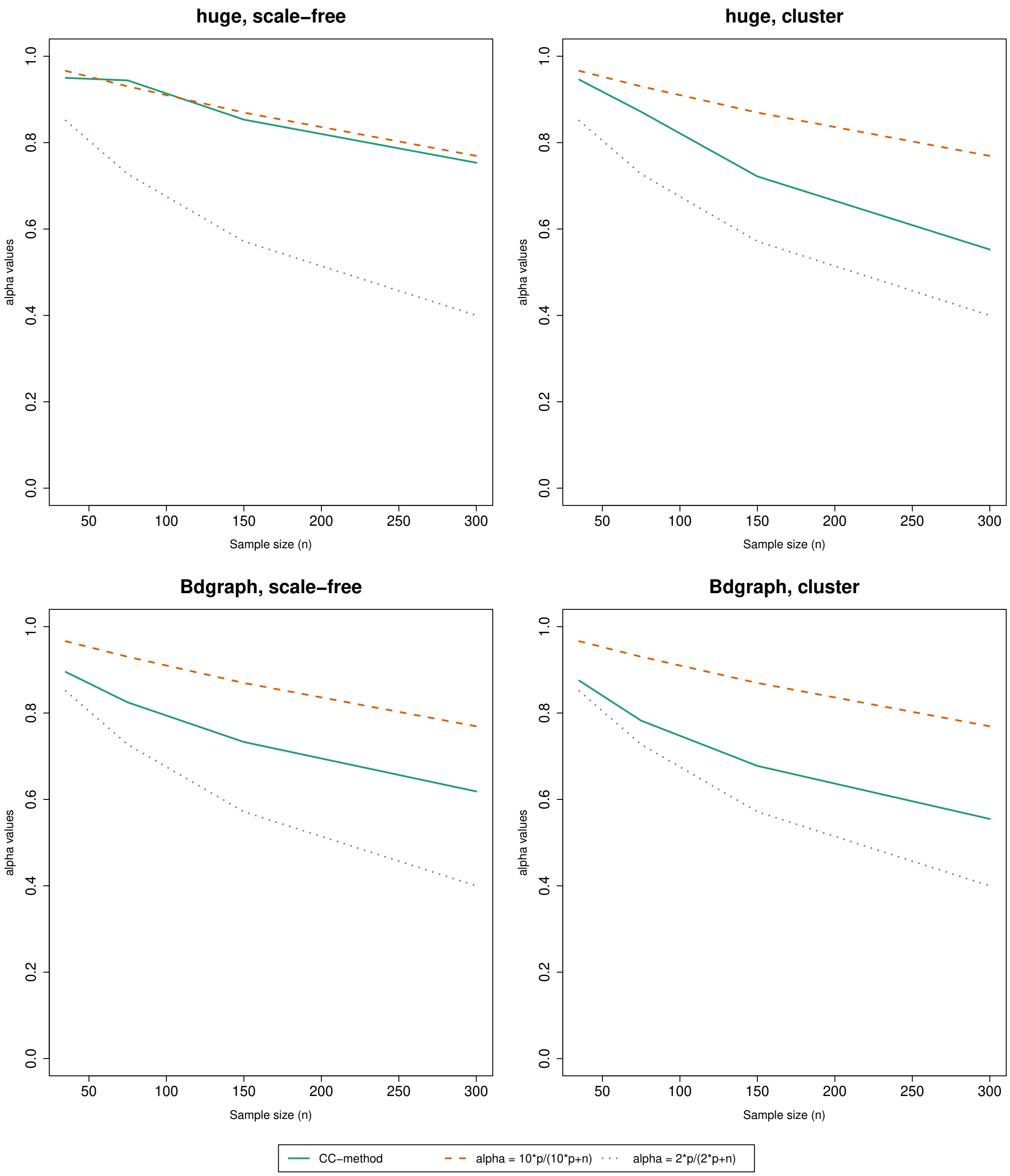

Fig E: Mean α values received with the CC-method. For reference, values with $\alpha = 10 \cdot p/(10 \cdot p + n)$ and $\alpha = 2 \cdot p/(2 \cdot p + n)$ are included.

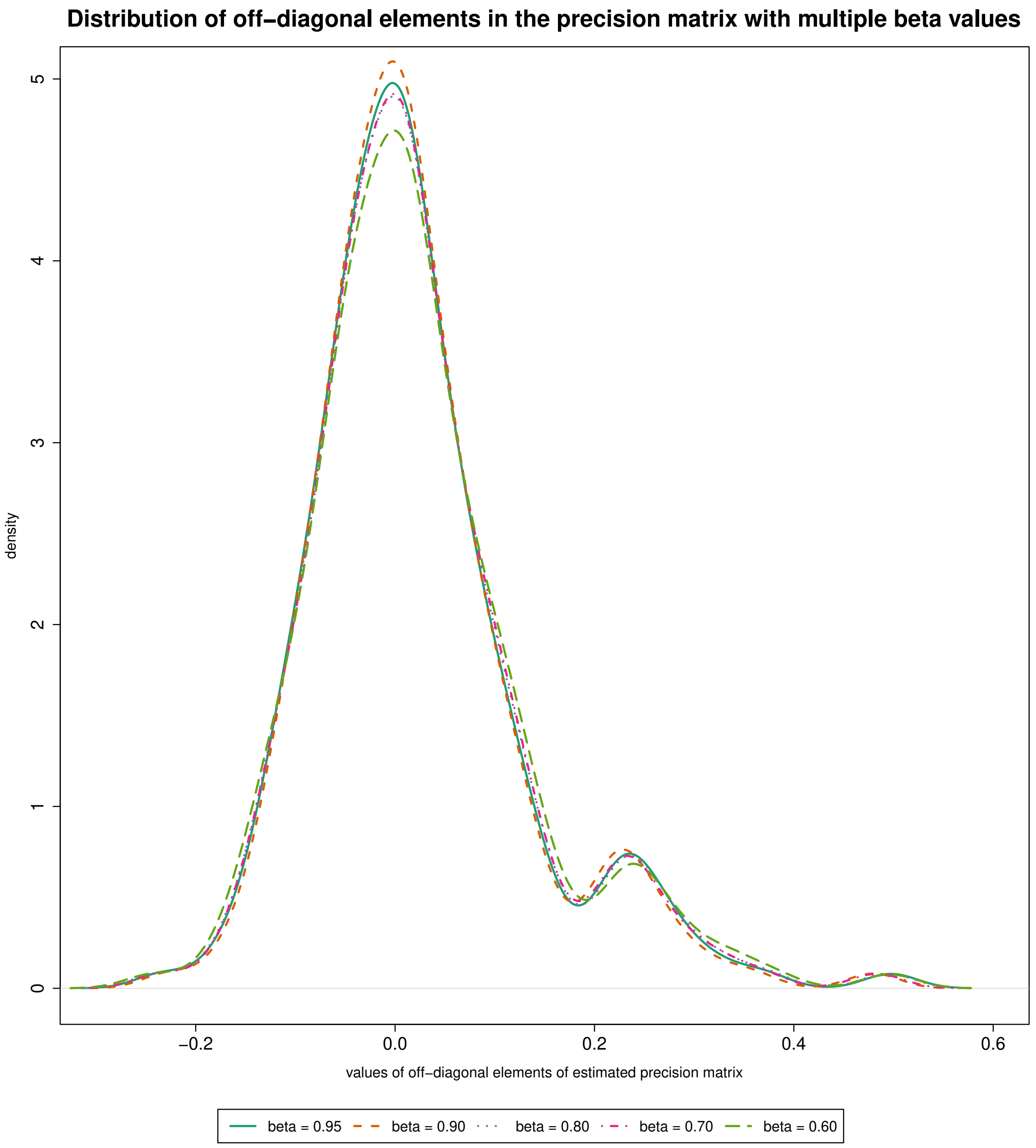

Fig F: Distribution of off-diagonal elements in the estimated precision matrix with multiple different β values. The α values are calculated individually for all β values using the CC-method. The graphical model considered in this example is a scale-free network generated with the R package `huge` $(n = 100, p = 20)$.

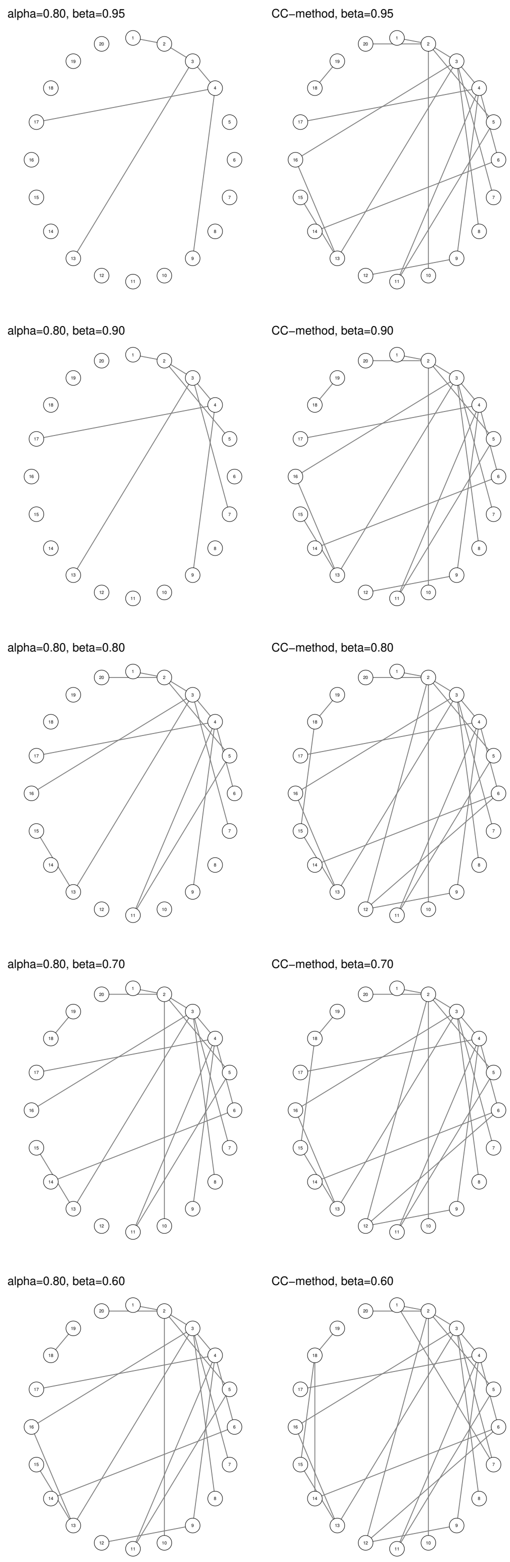

Fig G: Recovered networks with multiple β values. On the left, networks are estimated with $\alpha = 0.80$ and on the right, the networks are estimated with α values selected using the CC-method. Edges are selected using 90% credible intervals. The graphical model considered in this example is a scale-free network generated with the R package `huge` ($n = 100, p = 20$).

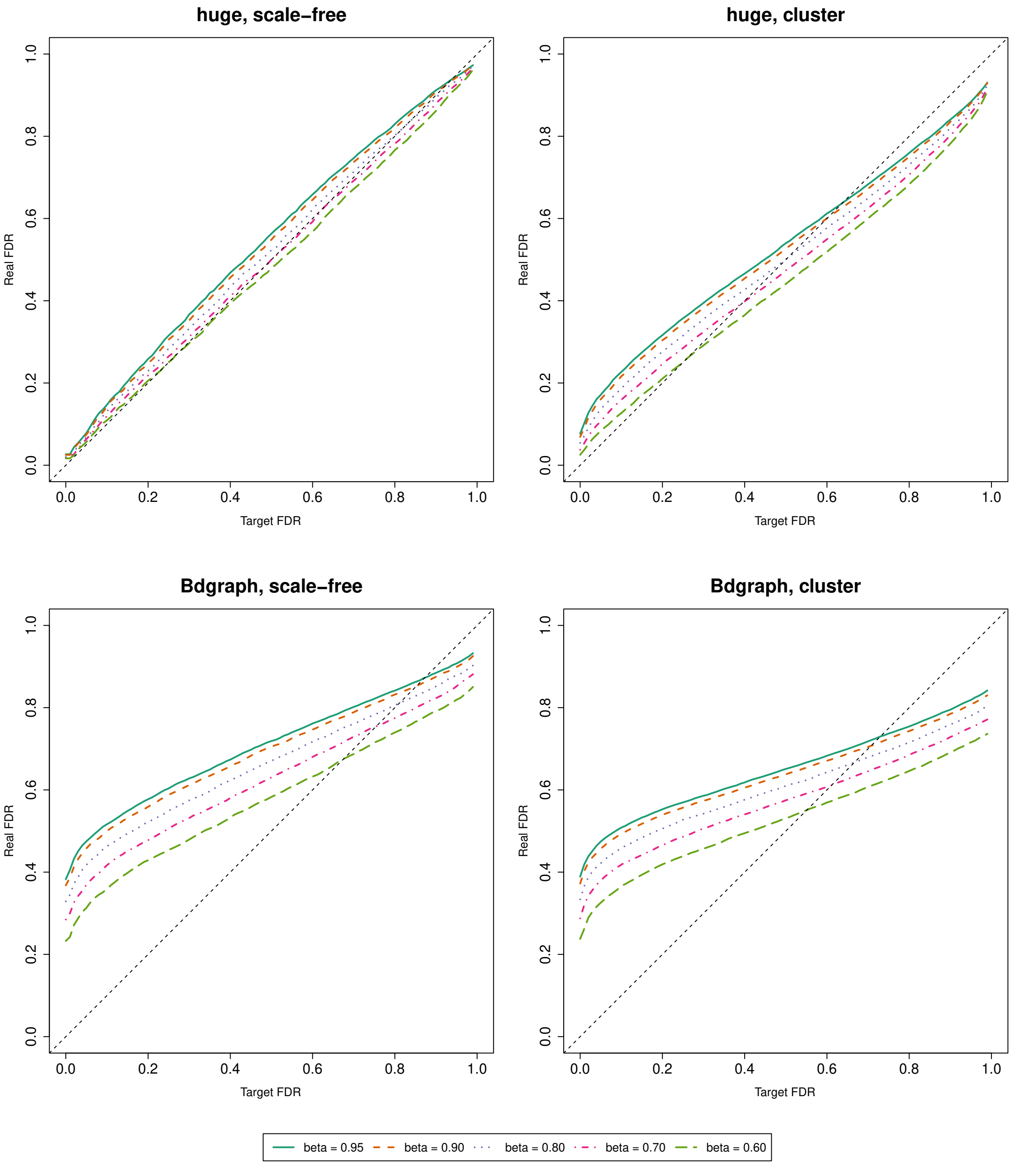

Fig H: Controlling FDR with multiple different β values. Averages of 50 different simulated datasets. For all datasets, $p = 100$ and $n = 300$. For all β values, $\alpha = 0.8$

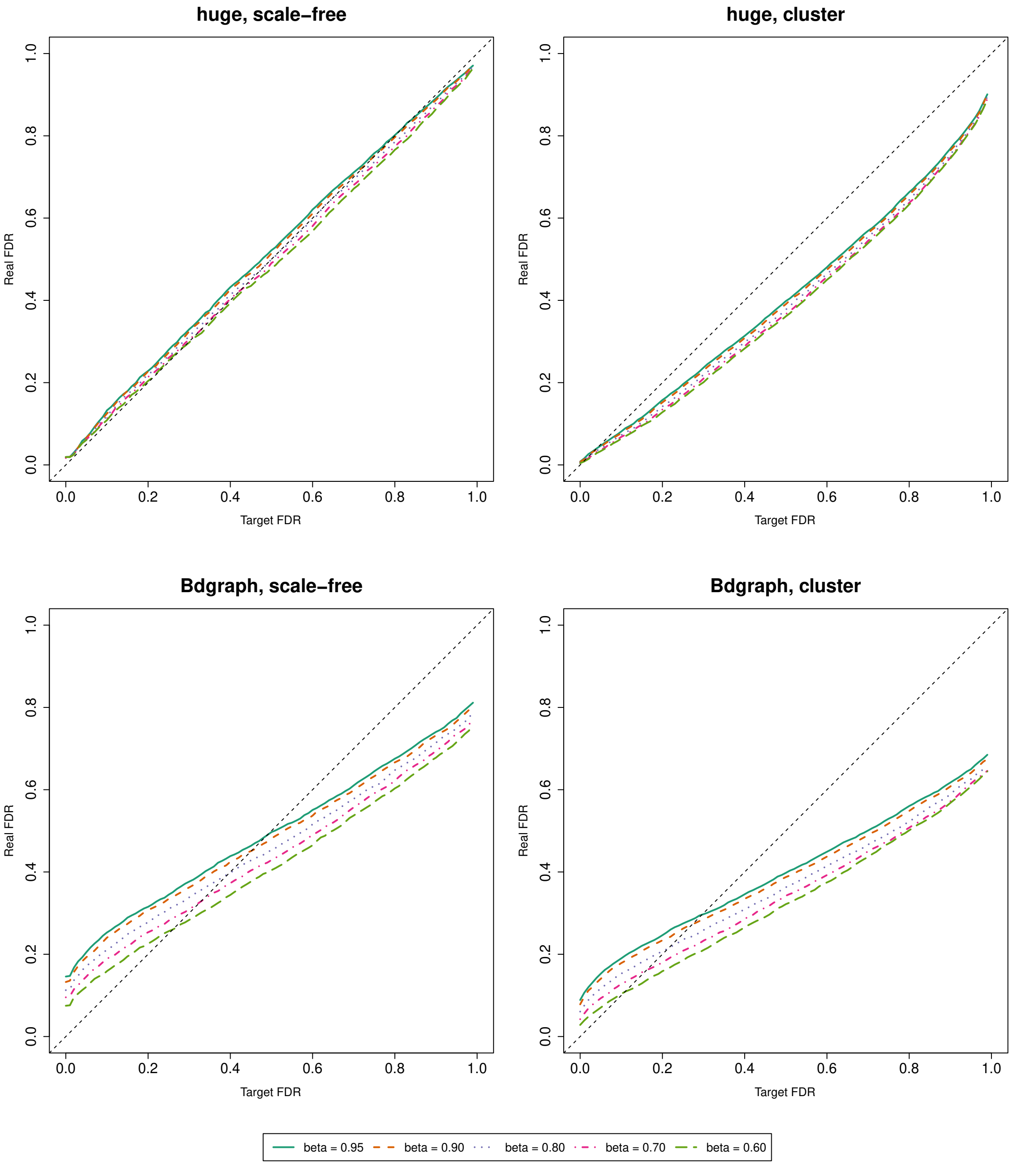

Fig I: Controlling FDR with multiple different β values. Averages of 50 different simulated datasets. For all datasets, $p = 100$ and $n = 300$. α value was selected for each β values individually using the CC-method.

F Variance-Gamma distribution's relation to off-diagonal elements

For the Wishart distribution, the marginal distribution for off-diagonal elements is known to be the Variance-Gamma (VG) distribution for a two-dimensional situation, which could be extended for higher dimensions (Fischer et al., 2025). This is expressed as,

$$X_{ij} \sim VG(n, v_{ij}, \sqrt{v_{ii}v_{jj} - v_{ij}^2}, 0),$$
$$\textit{if } X \sim W(n, V) \textit{ and } i \neq j.$$

Based on the fact that the full conditional posterior distribution for off-diagonal elements of Ω also follows the Wishart distribution, we could use the VG distribution as an approximation for the off-diagonal elements. We will still use the normal approximation for simplicity and computational ease. Also, the approximation is accurate enough as shown in the article Figs 3 and 4.

G The optimal credible interval

The estimated number of true positives $\widehat{\text{TP}}$ can further be limited to a predetermined expected graph size (K):

$$\widehat{\text{TP}} = \min\left(K, \left(\sum_{i<j} \hat{a}_{ij(\widehat{\text{CI}}(\gamma))} - \widehat{\text{FP}}\right)\right),$$

and false negatives can also be limited to nonnegative values, i.e.,

$$\widehat{\text{FN}} = \max\left(0, \left(K - \widehat{\text{TP}}\right)\right).$$

We choose not to use these approximations for FN and TP, because then the prior guess for graph size will become dominant.

H Measuring model accuracy

There are many ways of measuring model accuracy in classification problems, and it is important to choose the right metric such that the comparison between models

is meaningful. MCC, or Matthew's correlation coefficient, is known to be a better measurement value than F_1-score or balanced accuracy if the rightful classification of positives and negatives is equally important (Chicco et al., 2021). If positives are more important, then the F_1-score can be more informative, and F_1 favors denser networks compared to MCC.

MCC is defined as:

$$\mathrm{MCC} = \frac{\mathrm{TP}\cdot\mathrm{TN} - \mathrm{FP}\cdot\mathrm{FN}}{\sqrt{(\mathrm{TP}+\mathrm{FP})(\mathrm{TP}+\mathrm{FN})(\mathrm{TN}+\mathrm{FP})(\mathrm{TN}+\mathrm{FN})}},$$

where TP means true positives, TN true negatives, FP false positives, and FN false negatives.
F_1-score is calculated using TP, FP, and FN,

$$\mathrm{F}_1 = \frac{2\cdot\mathrm{TP}}{2\cdot\mathrm{TP}+\mathrm{FP}+\mathrm{FN}}.$$

F_1 values range from 0 to 1, and the MCC from -1 to 1, and a higher number indicates a better model accuracy.

The false discovery rate tells how large a portion of the estimated connections are false connections:

$$\mathrm{FDR} = \frac{\mathrm{FP}}{\mathrm{TP}+\mathrm{FN}} = \frac{\mathrm{FP}}{\mathrm{P}},$$

where P means the number of positives (or connections) in the real network. FDR ranges from 0 to 1, and smaller values are desirable.

The next metric to be used in this article is the true positive rate, which tells how larger a proportion of the true connections the method finds:

$$\mathrm{TPR} = \frac{\mathrm{TP}}{\mathrm{P}}.$$

TPR values range from 0 to 1, and a high value means that a larger proportion of positives are found; a value of 1 means that all of them are identified correctly.

For cluster-type networks, previous metrics are not as interesting. For clusters, different performance metrics have been developed that are able to measure the accuracy of recovering the correct clusters from the data and correctly identifying which variables belong to which cluster. We will use two metrics for this. The first is an average clustering coefficient (Newman, 2018). The Clustering coefficient CC_i for a node i tells how many of its neighbors are neighbors to each other, i.e., how many

nodes that are connected to the node i are connected to each other. If $\mathrm{CC}_i = 1$, then all neighbors are interconnected. If $\mathrm{CC}_i = 0$, then none of node i's neighbors are connected to node i's other neighbors. The clustering coefficient is defined as:

$$\mathrm{CC}_i = \frac{\sum_h \sum_j A_{ij} A_{jh} A_{hi}}{(\sum_j A_{ij})(\sum_j A_{ij} - 1)},$$

where A is an adjacency matrix. The clustering coefficient is calculated for each node individually, but the same idea can be extended to the whole graph. The average clustering coefficient, or ACC, is simply the average of all nodes' clustering coefficients:

$$\mathrm{ACC} = \frac{1}{p} \sum_i \mathrm{CC}_i,$$

where p is the number of nodes, or the number of variables. ACC describes how clustered the graph is in general.

For the last metric, we selected a normalized mutual information (NMI) (Kuncheva and Hadjitodorov, 2004; Danon et al., 2005), which is useful for measuring model accuracy for clustering purposes (Kuismin et al., 2021). It measures how much two clustering partitions (A,B) have in common. NMI is defined as:

$$\mathrm{NMI}(A, B) = \frac{-2 \sum_{i=1}^{c_A} \sum_{j=1}^{c_B} p_{ij} \log(\frac{p p_{ij}}{p_{.j} p_{i.}})}{\sum_{i=1}^{c_A} p_{i.} \log(\frac{p_{i.}}{p}) + \sum_{j=1}^{c_B} p_{.j} \log(\frac{p_{.j}}{p})},$$

where c_A and c_B are the number of clusters in partition A and B respectively. The $p_{i.}$, $p_{.j}$ and p_{ij} represent the number of nodes in partition A and i-th cluster, partition B and j-th cluster and both partitions (A and B) and clusters i and j, respectively. NMI ranges between 0 and 1. When NMI is 1, the partitions are the same (all clusters have the same members). If it is 0, the partitions have no common members.

I Technical details on reproducing the results

- The results for HMFGraph (optimal CI) were produced with the GEM algorithm, the α selected with the CC-method, and the optimal CI acquired with permutations and maximizing the estimated F_1-score (expected number of connections is set to the number of variables p). Using the R package `HMFGraph`, this is done with function *HMFGraph* or with functions *HMFGraph_ GEM*, *HMFGraph_ GEM_permutations* and *HMFGraph_ GEM_ optimal_ CI* (default parameter values) (the example 1 at `https://github.com/AapoKorhonen/HMFGraph`) (1.0 version, October 21, 2025).

- The results for HMFGraph (target FDR = 0.20) were produced with the GEM algorithm, the α selected with the CC-method. The CI was selected using Equation (27) in this article ($\text{FDR}_{\text{target}} = 0.20$), with 50 permutations. Using the `HMFGraph` package, this is done with functions *HMFGraph_GEM*, *HMFGraph_GEM_permutations* and *HMFGraph_GEM_FDR_control* (default parameter values) (the example 2 at `https://github.com/AapoKorhonen/HMFGraph`) (1.0 version, October 21, 2025).

- Glasso with StARS was run with the R package `pulsar` using functions called *pulsar* and *refit*. The functions were used following the first example provided in the R package `pulsar` documentation `https://cran.r-project.org/web/packages/pulsar/vignettes/pulsar.html` (Version: 0.3.11). All the same parameter values were used as in their example, except the method was set to "glasso".

- R package `beam` can be downloaded from Github page at `https://github.com/gleday/beam` (We used Version: 2.0.2). The results were produced with functions *beam* and *beam.select* and the graph structure recovered with the function *ugraph*. All default parameters were used.

- The results for the method CLEVEL were attained by using the R package `PCGII` and with functions *clevel* and *inference* (R package version 1.1.2) (Wang et al., 2024). We followed the documented example from the function *clevel* documentation. All default parameter values were used, and additionally, both 0.05 and 0.20 target FDR values (parameter alpha in the function *clevel*, 0.05 is the default value).

- The results for thAV were attained with the R package `thav.glasso` `https://github.com/MikeLasz/thav.glasso` (4.4.2025). The package was downloaded from another GitHub page, because the original had a documentation error that led to an installation error (`https://github.com/apatrone2/thav.glasso`). The package was used following the example on the original GitHub page using the function *thAV.estimator*. All default parameters were used.

- The method TIGER is implemented in the R package `flare` (Version 1.7.0.1) (Li et al., 2015). The results were attained with the function *sugm*. All default parameters were used.

- The G-Wishart is implemented in the R package `BDgraph` (Version: 2.72). The results were attained with the function *bdgraph* and the adjacency matrix acquired from the *bdgraph.obj$p_links* object (if the posterior probability

is > 0.5, then the corresponding adjacency matrix value is 1). All default parameter values were used (including the "bdmcmc" algorithm).

- BGGM was tested with the R package `BGGM` (version 2.1.5). We used the functions *estimate* and *select* with default parameter values following the example on the package's GitHub page `https://donaldrwilliams.github.io/BGGM/`.

Additionally, all HMFGraph results were run with $\alpha = 10 \cdot p/(10 \cdot p + n)$ with the cluster network datasets.

J Comparisons of network recovery

Next, we showcase how the recovered networks differ between each tested GGM method. The datasets considered in this example are simulated with the `huge` and `BDgraph` R packages. Scale-free and cluster network types are included. The true networks and recovered networks for each method are shown in Figs J, K, L, and M.

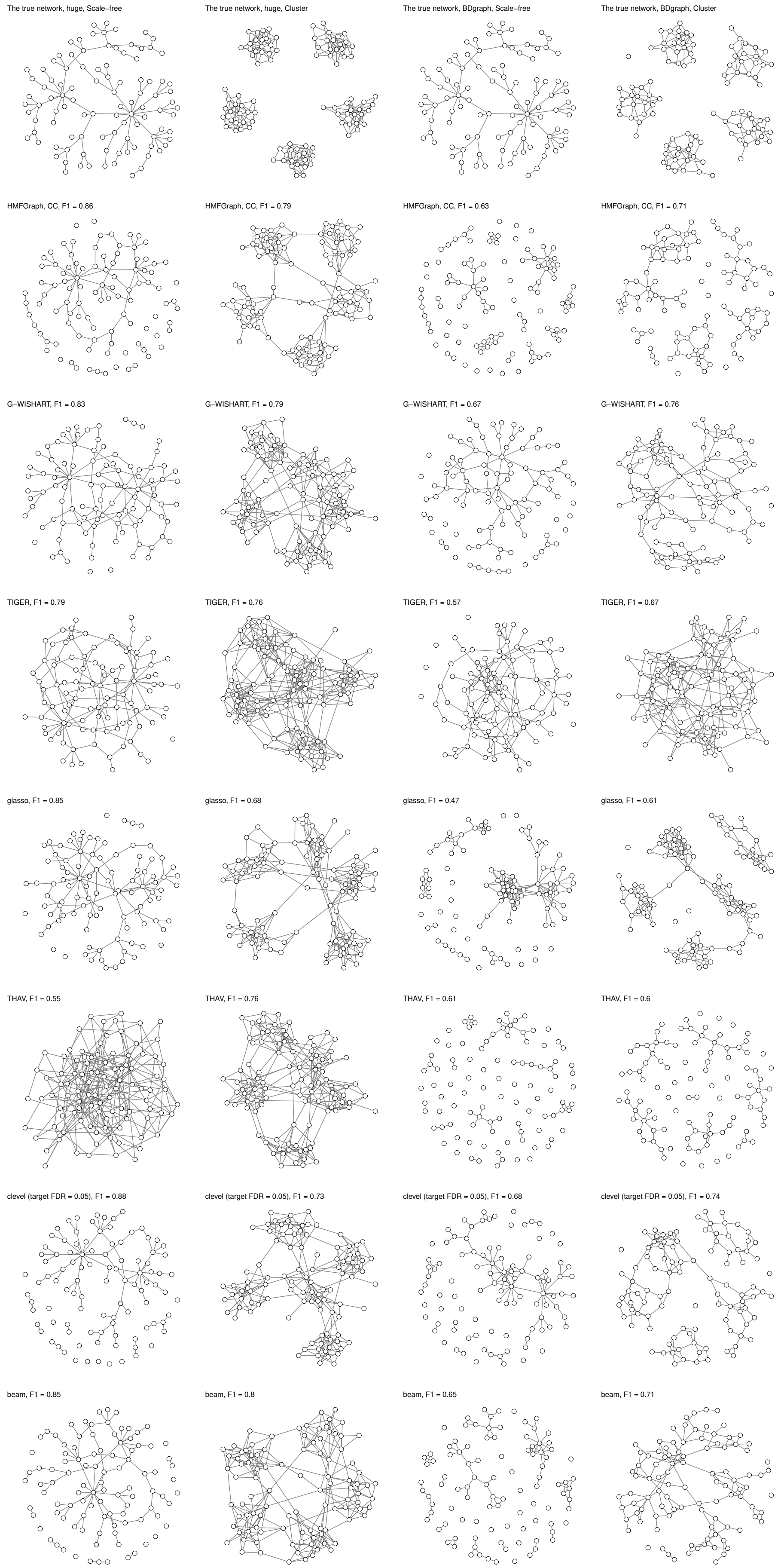

Fig J: Comparison of recovered networks with multiple GGMs methods ($n = 300, p = 100$). The true networks are illustrated in the first row.

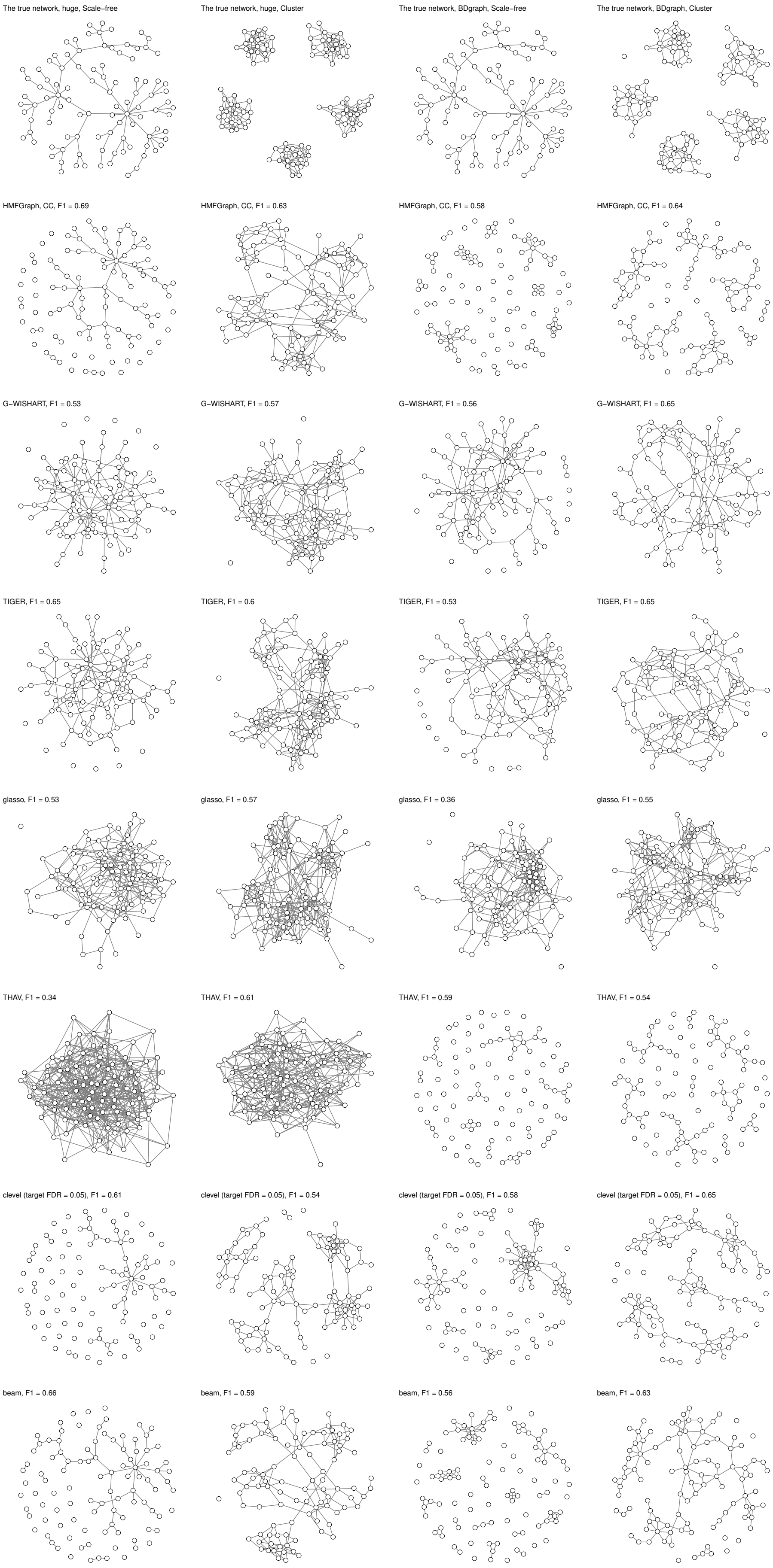

Fig K: Comparison of recovered networks with multiple GGMs methods ($n = 150, p = 100$). The true networks are illustrated in the first row.

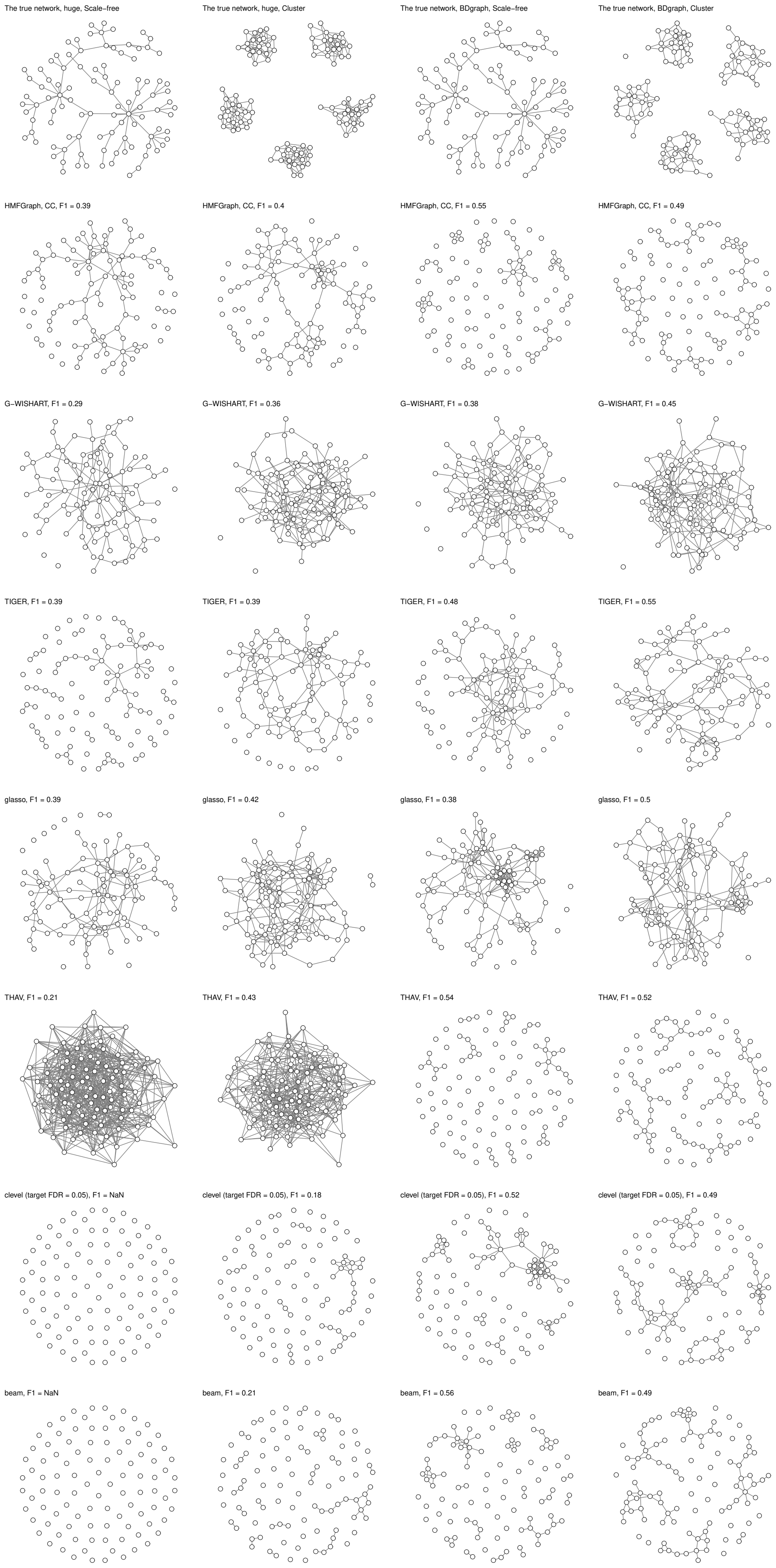

Fig L: Comparison of recovered networks with multiple GGMs methods ($n = 75, p = 100$). The true networks are illustrated in the first row.

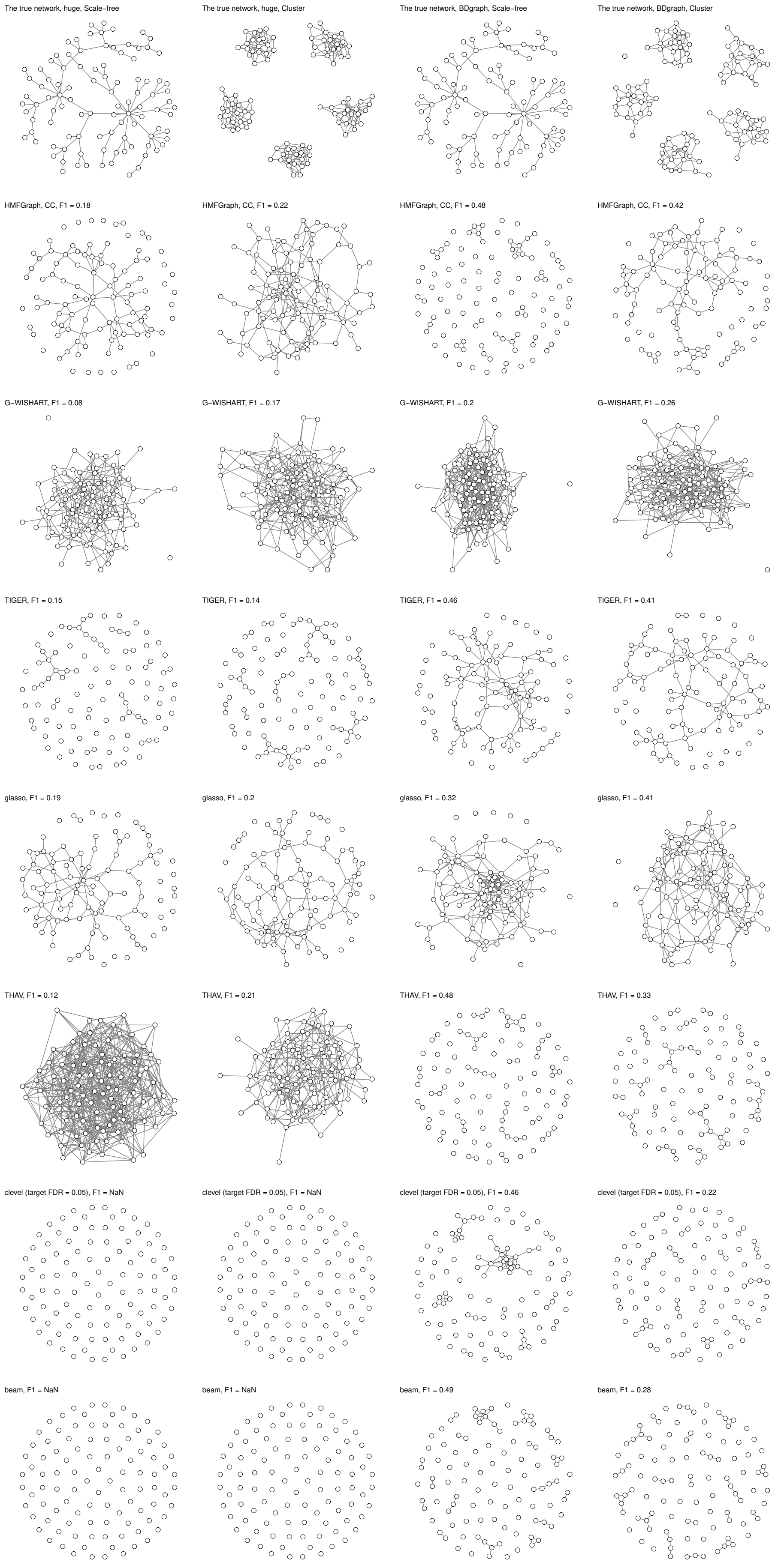

Fig M: Comparison of recovered networks with multiple GGMs methods ($n = 35, p = 100$). The true networks are illustrated in the first row.

K Riboflavin dataset

In Fig N, with target FDR = 0, we can clearly see a cluster that includes nodes 42, 95-99, and 101. The genes corresponding to those nodes are: SIGY_at, YXLC_at, YXLD_at, YXLE_at, YXLF_at, YXLG_at, and YXLJ_at.

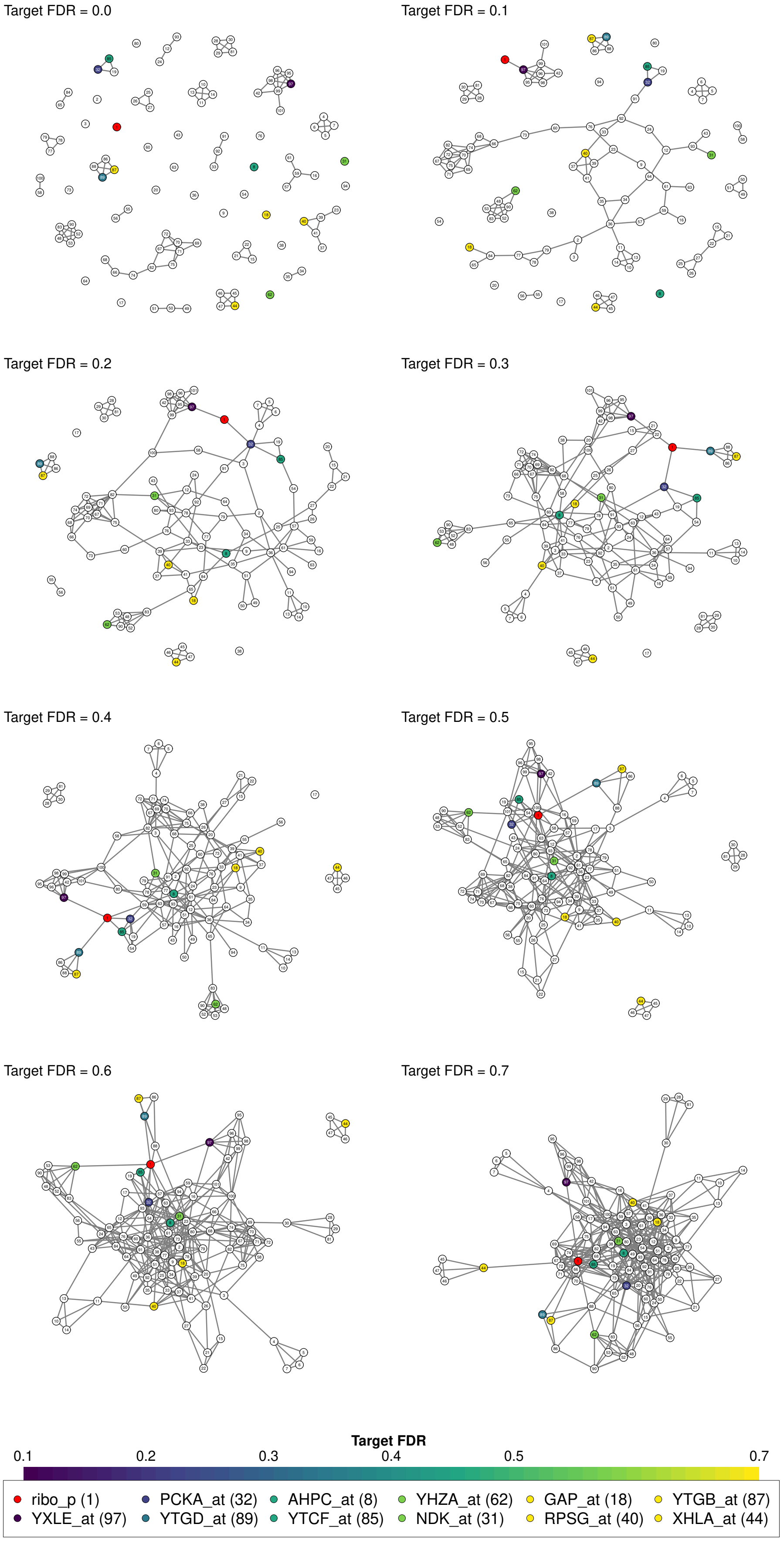

Fig N: Constructed networks from the riboflavin dataset with multiple target FDR values. The node describing the riboflavin production is highlighted in red.

L American gut data

We noticed with the gut dataset result that with a large α value (Fig O B and Fig P B), all connections indicate a positive partial correlation. This means that if two nodes (OTUs) are connected, they are positively partially correlated. When the optimal values for α were selected with the CC-method, some connections indicate a negative partial correlation.

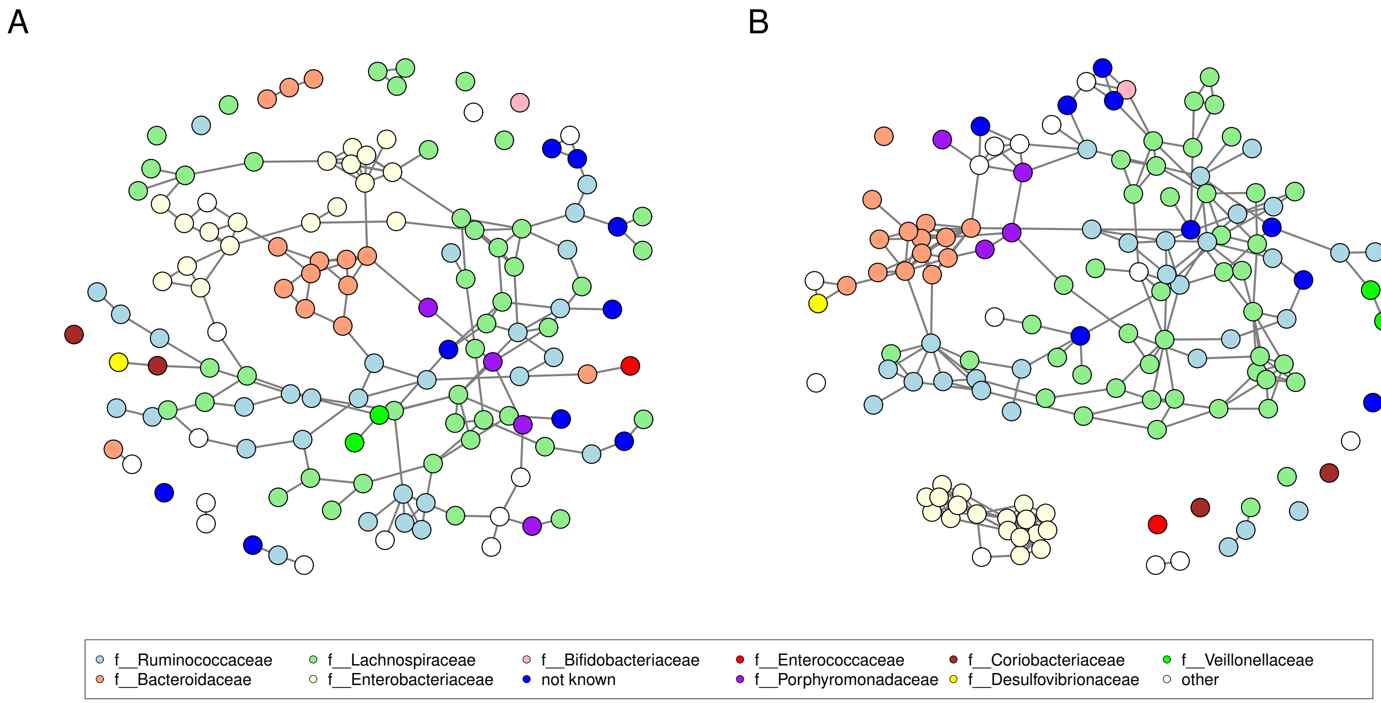

Fig O: The network recovered from the gut data set using HMFGraph. An optimal credible interval was selected using 50 permutations. Different OTU groups (in *family* taxonomic rank) are illustrated with different colors. **(A)** An optimal value of parameter α was selected based on the CC-method ($\alpha \approx 0.39$). **(B)** The value of parameter α was set to be large (e.g., $\alpha = 10 \cdot p/(10 \cdot p + n) \approx 0.82$).

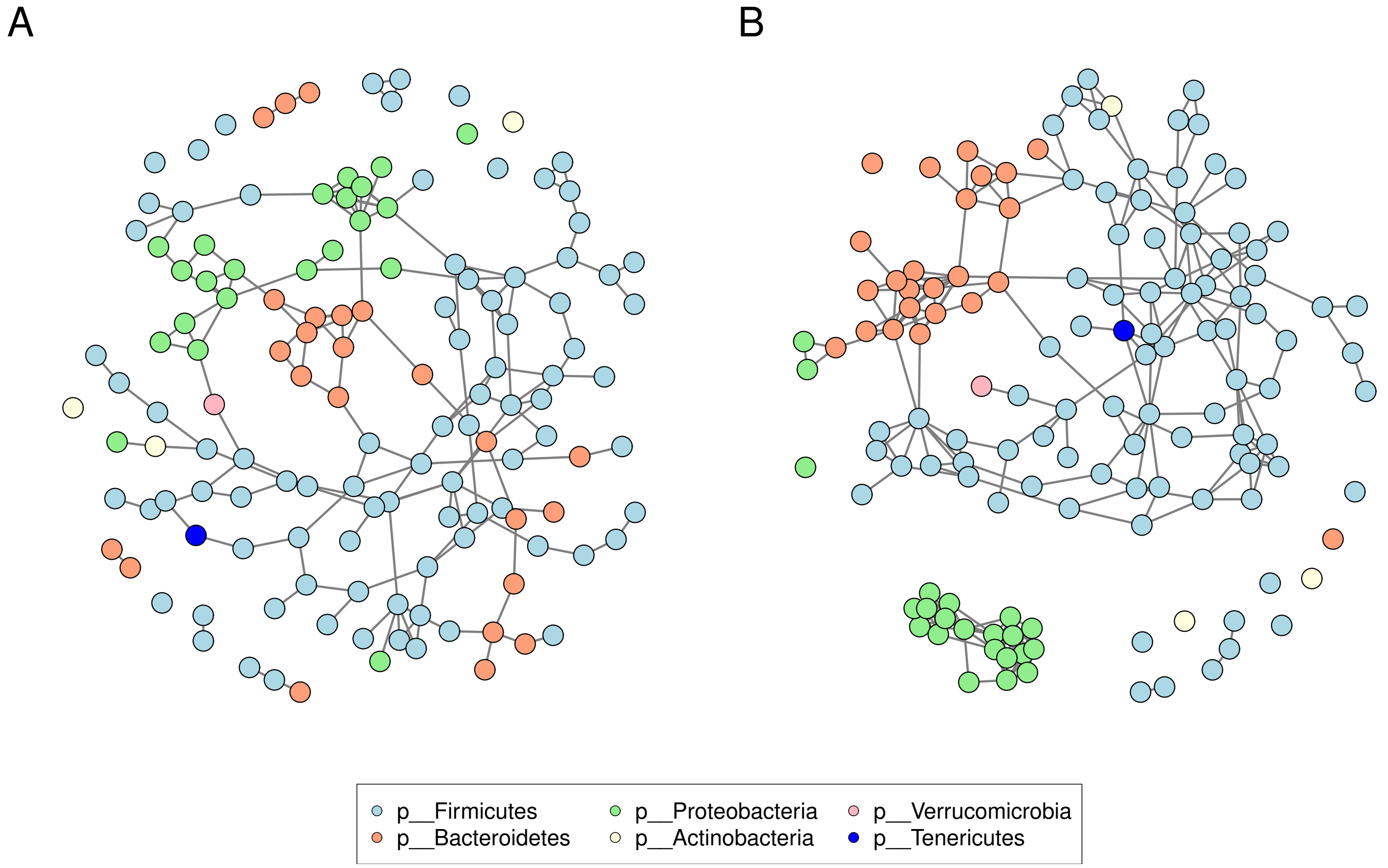

Fig P: The network recovered from the gut data set using HMFGraph. An optimal credible interval was selected using 50 permutations. Different OTU groups (in *phyla* taxonomic rank) are illustrated with different colors. **(A)** An optimal value of parameter α was selected based on the CC-method ($\alpha \approx 0.39$). **(B)** The value of parameter α was set to be large (e.g., $\alpha = 10 \cdot p/(10 \cdot p + n) \approx 0.82$).

M Proof of proper posterior distribution: improper prior

The posterior distribution has to be proper, i.e.,

$$\int p(\Pi|Y)d\Pi < \infty,$$

where $\Pi = (\Omega, \Phi, b_{11}, b_{22}, \ldots, b_{pp})$ denotes all the model parameters.

Based on Fubini's theorem, we can change the order of integration. Firstly, we integrate out each diagonal element of the matrix B. From Equation (J) we know that the full conditional distribution for each b_{ii} is gamma distribution:

$$p(b_{ii}|\cdot) \propto b_{ii}^{\frac{\delta+p-1}{2}-1} \exp\left[-\frac{1}{2}b_{ii}(\delta+p-1)\phi_{ii}\right].$$

Therefore each integration with respect to b_{ii} is

$$\int p(b_{ii}|\cdot)db_{ii} \propto \int b_{ii}^{\frac{\delta+p-1}{2}-1} \exp\left[-\frac{1}{2}b_{ii}(\delta+p-1)\phi_{ii}\right] db_{ii}$$
$$= \left(\frac{\delta+p-1}{2}\phi_{ii}\right)^{-\frac{\delta+p-1}{2}} \Gamma(\delta+p-1),$$

where ϕ_{ii} is a diagonal element of matrix Φ. If all b_{ii} are individually integrated, then the posterior distribution changes to

$$\int \cdots \int p(b_{11}, b_{22}, \ldots, b_{ii}|\cdot)db_{11}\cdots db_{pp} \propto \prod_{i=1}^{p}\left(\frac{\delta+p-1}{2}\phi_{ii}\right)^{-\frac{\delta+p-1}{2}} \Gamma(\delta+p-1)$$
$$= \left(\left(\frac{\delta+p-1}{2}\right)^{-\frac{\delta+p-1}{2}} \Gamma(\delta+p-1)\right)^{p} \prod_{i=1}^{p}(\phi_{ii})^{-\frac{\delta+p-1}{2}} \propto \prod_{i=1}^{p}(\phi_{ii})^{-\frac{\delta+p-1}{2}}. \tag{S}$$

Hadamard's inequality (Różański et al., 2017) states that if a matrix A is a positive-definite matrix, then:

$$|A| \leq \prod_{i=1}^{p} a_{ii}. \tag{T}$$

Because Φ is a positive-definite matrix, we can now apply Equations (S) and (T) to state that:

$$\prod_{i=1}^{p}(\phi_{ii})^{-\frac{\delta+p-1}{2}} \leq |\Phi|^{-\frac{\delta+p-1}{2}} \tag{U}$$

Secondly, we integrate with respect to the parameter Φ. This becomes straightforward thanks to the inequality (U). From Equations (B), (C) and (S) we get:

$$\int p(\Omega, \Phi|Y)d\Phi$$
$$\propto \int |(\nu-p-1)\Phi|^{\frac{\nu}{2}} |\Phi|^{\frac{\delta+p-1-p-1}{2}} \prod_{i=1}^{p}(\phi_{ii})^{-\frac{\delta+p-1}{2}} \exp\left[-\frac{1}{2}\mathrm{tr}((\nu-p-1)\Phi\Omega)\right] d\Phi$$
$$\leq \int |(\nu-p-1)\Phi|^{\frac{\nu}{2}} |\Phi|^{\frac{\delta+p-1-p-1}{2}} |\Phi|^{-\frac{\delta+p-1}{2}} \exp\left[-\frac{1}{2}\mathrm{tr}((\nu-p-1)\Phi\Omega)\right] d\Phi$$
$$\propto \int |\Phi|^{\frac{\nu-p-1}{2}} \exp\left[-\frac{1}{2}\mathrm{tr}((\nu-p-1)\Phi\Omega)\right] d\Phi,$$

where

$$|\Phi|^{\frac{\nu-p-1}{2}} \exp\left[-\frac{1}{2}\mathrm{tr}((\nu-p-1)\Phi\Omega)\right] \propto W(\Phi; \nu, ((\nu-p-1)\Omega)^{-1}),$$

and therefore the integral is

$$\int |\Phi|^{\frac{\nu-p-1}{2}} \exp\left[-\frac{1}{2}\mathrm{tr}((\nu-p-1)\Phi\Omega)\right] d\Phi$$
$$\propto 2^{\frac{\nu p}{2}} |((\nu-p-1)\Omega)^{-1}|^{\frac{\nu}{2}} \Gamma_p\left(\frac{\nu}{2}\right)$$
$$\propto |((\nu-p-1)\Omega)^{-1}|^{\frac{\nu}{2}} \propto |\Omega^{-1}|^{\frac{\nu}{2}}. \tag{V}$$

Lastly, we integrate with respect to the parameter Ω. Combining Equations (A), (B) and (V) we finally get:

$$\int p(\Omega|Y)d\Omega \propto \int |\Omega|^{\frac{n}{2}} |\Omega|^{\frac{\nu-p-1}{2}} |\Omega^{-1}|^{\frac{\nu}{2}} \exp\left[-\frac{1}{2}\mathrm{tr}(nS\Omega)\right] d\Omega$$
$$= \int |\Omega|^{\frac{n-p-1}{2}} \exp\left[-\frac{1}{2}\mathrm{tr}(nS\Omega)\right] d\Omega,$$

where

$$|\Omega|^{\frac{n-p-1}{2}} \exp\left[-\frac{1}{2}\mathrm{tr}(nS\Omega)\right] \propto W(\Omega; n, (nS)^{-1})),$$

which can also be stated as $IW(\Sigma; n, nS)$, which is a proper density function if $n > p-1$ and nS is positive-definite matrix (only possible when $n > p-1$):

$$\int |\Omega|^{\frac{n-p-1}{2}} \exp\left[-\frac{1}{2}\mathrm{tr}(nS\Omega)\right] d\Omega < \infty.$$

Thus, the posterior distribution is proper, at least when $n > p-1$.

N Proof of proper posterior distribution: gamma prior

As seen in the previous section, the improper prior (F) produces a proper posterior function if $n > p - 1$. In this section, we investigate whether the gamma prior (D) leads to a proper posterior density even when $n < p$.

Again, the posterior distribution is proper if

$$\int p(\Pi|Y)d\Pi < \infty,$$

where $\Pi = (\Omega, \Phi, b_{11}, b_{22}, \dots, b_{pp})$ denotes all the model parameters.

Based on Fubini's theorem, we can change the order of integration. This time, we start with Ω. Because the full conditional distribution is known (see Equation (G)), the integral is simply

$$\begin{aligned}
&\int p(\Omega, b_{11}, \dots, b_{pp}, \Phi|S)d\Omega \\
&\propto \int |\Omega|^{\frac{\nu+n-p-1}{2}} \exp\left[-\frac{1}{2}\mathrm{tr}((nS + (\nu-p-1)\Phi)\Omega)\right] d\Omega \\
&\propto \frac{1}{|nS + (\nu-p-2)\Phi|^{\frac{\nu+n}{2}}}.
\end{aligned} \tag{W}$$

If A and B are positive-semidefinite, then the following inequality holds (Paksoy et al., 2014):

$$|A + B| \geq |A| + |B|. \tag{X}$$

Because Φ and nS are always positive-semidefinite (even if $n < p$), we can simplify (W) using the inequality (X) to:

$$\begin{aligned}
&\frac{1}{|(nS + (\nu-p-2)\Phi)|^{\frac{\nu+n}{2}}} \leq \frac{1}{(|nS| + |(\nu-p-2)\Phi)|)^{\frac{\nu+n}{2}}} \leq \frac{1}{(|(\nu-p-2)\Phi)|)^{\frac{\nu+n}{2}}} \\
&= |(\nu-p-2)\Phi|^{-\frac{\nu+n}{2}} \propto |\Phi|^{-\frac{\nu+n}{2}}.
\end{aligned} \tag{Y}$$

Now we integrate with respect to the parameter Φ. Using the inequality (Y), and

combining (C), (B) and (W) the integral simplifies to:

$$\begin{aligned}
&\int p(\Phi, b_{11}, \ldots, b_{pp}|Y) d\Phi \\
&\propto \int \frac{1}{|(nS + (\nu - p - 2)\Phi)|^{\frac{\nu+n}{2}}} |\Phi|^{\frac{\delta+p-1-p-1}{2}} |\Phi|^{\frac{\nu}{2}} \exp\left[-\frac{1}{2}\mathrm{tr}((\delta + p - 1)B\Phi)\right] d\Phi \\
&\leq \int |(\nu - p - 2)\Phi|^{-\frac{\nu+n}{2}} |\Phi|^{\frac{\delta+p-1-p-1}{2}} |\Phi|^{\frac{\nu}{2}} \exp\left[-\frac{1}{2}\mathrm{tr}((\delta + p - 1)B\Phi)\right] d\Phi \\
&\propto \int |\Phi|^{-\frac{\nu+n}{2}} |\Phi|^{\frac{\delta+p-1-p-1}{2}} |\Phi|^{\frac{\nu}{2}} \exp\left[-\frac{1}{2}\mathrm{tr}((\delta + p - 1)B\Phi)\right] d\Phi \\
&\propto \int |\Phi|^{\frac{\delta+p-n-1-p-1}{2}} \exp\left[-\frac{1}{2}\mathrm{tr}((\delta + p - 1)B\Phi)\right] d\Phi,
\end{aligned}$$

where

$$|\Phi|^{\frac{\delta+p-n-1-p-1}{2}} \exp\left[-\frac{1}{2}\mathrm{tr}((\delta + p - 1)B\Phi)\right] \propto W(\Phi; (\delta+p-n-1); ((\delta+p-1)B)^{-1}),$$

and thus the integral is

$$\begin{aligned}
&\int |\Phi|^{\frac{\delta+p-n-1-p-1}{2}} \exp\left[-\frac{1}{2}\mathrm{tr}((\delta + p - 1)B\Phi)\right] d\Phi \\
&\propto |((\delta + p - 1)B)^{-1}|^{\frac{\delta-n+p-1}{2}} \\
&\propto |B|^{-\frac{\delta-n+p-1}{2}}. \qquad \text{(Z)}
\end{aligned}$$

Lastly, we integrate with respect to the parameter b_{ii}. From Equations (Z), (C) and (E) we get:

$$\begin{aligned}
&\int p(b_{ii}|\cdot) db_{ii} \\
&\propto \int_0^\infty |B|^{-\frac{\delta-n+p-1}{2}} |B|^{\frac{\delta+p-1}{2}} \frac{\epsilon_2^{\epsilon_1}}{\Gamma(\epsilon_1)} b_{ii}^{\epsilon_1 - 1} \exp\left[-\epsilon_2 b_{ii}\right] db_{ii} \\
&\propto \int_0^\infty b_{ii}^{\frac{n}{2}+\epsilon_1-1} \exp\left[-\epsilon_2 b_{ii}\right] db_{ii}.
\end{aligned}$$

Now,

$$b_{ii}^{\frac{n}{2}+\epsilon_1-1} \exp\left[-\epsilon_2 b_{ii}\right] \propto \mathrm{Gamma}(\mathrm{shape} = \frac{n}{2} + \epsilon_1, \mathrm{rate} = \epsilon_2).$$

And this leads to:

$$\propto \int_0^\infty b_{ii}^{\frac{n}{2}+\epsilon_1-1} \exp\left[-\epsilon_2 b_{ii}\right] db_{ii} < \infty,$$

which is true for all b_{ii} (ϵ_1, $\epsilon_2 > 0$) and therefore the posterior is proper even when $n < p$.

References

D. Chicco, N. Tötsch, and G. Jurman. The Matthews correlation coefficient (MCC) is more reliable than balanced accuracy, bookmaker informedness, and markedness in two-class confusion matrix evaluation. *BioData Mining*, 14(1):1–22, 2021. doi: 10.1186/s13040-021-00244-z.

L. Danon, A. Diaz-Guilera, J. Duch, and A. Arenas. Comparing community structure identification. *Journal of statistical mechanics: Theory and experiment*, 2005: P09008, 2005.

A. Fischer, R. E. Gaunt, and A. Sarantsev. The Variance-Gamma distribution: A review. *Statistical Science*, 40(2):235–258, 2025. doi: 10.1214/24-STS929. URL `https://doi.org/10.1214/24-STS929`.

J. Kim, S. Kim, and S. Choi. Learning to warm-start bayesian hyperparameter optimization. *arXiv preprint*, 2018. doi: 10.48550/arXiv.1710.06219. URL `https://arxiv.org/abs/1710.06219`.

M. Kuismin, F. Dodangeh, and M. J. Sillanpää. Gap-com: general model selection criterion for sparse undirected gene networks with nontrivial community structure. *G3 (Bethesda)*, 12(2):jkab437, 12 2021. ISSN 2160-1836. doi: 10.1093/g3journal/jkab437.

L. I. Kuncheva and S. T. Hadjitodorov. Using diversity in cluster ensembles. In *2004 IEEE international conference on systems, man and cybernetics (IEEE Cat. No. 04CH37583)*, volume 2, pages 1214–1219. IEEE, 2004.

G. G. R. Leday and S. Richardson. Fast Bayesian inference in large Gaussian graphical models. *Biometrics*, 75(4):1288–1298, 04 2019. ISSN 0006-341X. doi: 10.1111/biom.13064.

O. Ledoit and M. Wolf. Honey, i shrunk the sample covariance matrix. *Journal of Portfolio Management*, 30(4):110–119, 2004a. doi: 10.3905/jpm.2004.110.

O. Ledoit and M. Wolf. A well-conditioned estimator for large-dimensional covariance matrices. *Journal of Multivariate Analysis*, 88(2):365–411, 2004b. ISSN 0047-259X. doi: 10.1016/S0047-259X(03)00096-4.

X. Li, T. Zhao, X. Yuan, and H. Liu. The flare package for high dimensional linear regression and precision matrix estimation in r. *Journal of Machine Learning Research*, 16(18):553–557, 2015.

M. Newman. *Networks*. Oxford University Press, 2018.

V. Paksoy, R. Turkmen, and F. Zhang. Inequalities of generalized matrix functions via tensor products. *The Electronic Journal of Linear Algebra*, 27:332–341, 2014.

M. Różański, R. Wituła, and E. Hetmaniok. More subtle versions of the hadamard inequality. *Linear Algebra and its Applications*, 532:500–511, 2017.

H. Wang, Y. Qiu, and P. Liu. *PCGII: Partial correlation graph with information incorporation*, 2024. URL `https://haowang47.github.io/PCGII/`. R package version 1.1.2.
