# Supplementary material for: HMFGraph: Novel Bayesian approach for recovering biological networks
Source: PLoS Comput Biol. 2025 Oct 30;21(10):e1013614. doi: 10.1371/journal.pcbi.1013614 (PMC12591435; doi:10.1371/journal.pcbi.1013614)
Supplement: Attachment — Submitted filename: Response_to_Reviewers.pdf [file pcbi.1013614.s002.pdf]

Detailed answers to reviewers' comments. PCOMPBIOL-D-25-00977 HMFGraph: Novel Bayesian approach for recovering biological networks PLOS Computational Biology

Korhonen et al.

September 2025

Reviewer 1

Comment 1: The abstract should clearly articulate the current limitations in existing approaches in order to highlight the novelty and contributions of this work.

Thank you for pointing this out. In the revised manuscript, we have modified the abstract to clearly point out some major limitations of previous methods. Usually, the most demanding parts of GGM implementations are: (i) hyperparameter tuning, (ii) edge selection, (iii) scalability for large datasets, and (iv) the prior choice for Bayesian GGM. Our proposed method addresses all these limitations. We feel that the revised abstract is now more informative than the original version.

Comment 2: The introduction is recommended to be enriched with more background on real-world biological applications of network recovery, such as cancer pathway inference or metabolic network reconstruction, in order to better demonstrate the practical relevance and appeal of the method.

In the revised manuscript, we added a new paragraph to the introduction section that gives background for biological networks. We also included more discussion on features common in biological data and how our method is well-suited for them.

Comment 3: While the proposed hierarchical Matrix-F prior is theoretically innovative, a more systematic and quantitative comparison with existing Matrix-F-based models is needed—particularly regarding stability and inference performance in high-dimensional settings.

We recognize that our original manuscript lacked comparisons with existing matrix-F implementations. The only current implementation of GGM using the Matrix-F prior is an R package called BGGM. The package is designed for psychological datasets, which often have a large number of samples and a low number of variables ($n > p$, or $n >> p$). The implementation does not work at all with high-dimensional data.

To address this problem, we decided to include results with BGGM in situations where $n \approx p$ (i.e., test situations where $n = 150, p = 100$ and $n = 300, p = 100$). From those results, we can see that our method has clearly better network recovery capabilities than the BGGM. This points out that the current implementation is not designed for high- or close-to-high-dimensional situations, and our method is a clear improvement.

Comment 4: The section on Gaussian graphical models reads more like a preliminaries/background section than a direct part of the model description. It might be clearer if repositioned or retitled accordingly.

We renamed some of the section titles to improve the manuscript's structure. The revised manuscript now has a clearer structure which is easier to follow.

Comment 5: The methodological presentation requires a major revision. Rather than listing only what modifications were made to the model, I would prefer a more structured exposition that traces the complete modeling pipeline—from input to output—with the innovations clearly embedded within that logical flow.

We have now improved the methodological part of the manuscript by changing the titles of the method section (see answer to comment 4) and by introducing a flow chart that showcases all important parts of the proposed GGM approach (see more on answer to comment 6). Additionally, we have now presented our proposed hierarchical matrix F prior as a whole in a separate section.

Comment 6: A high-level schematic or workflow diagram summarizing the modeling procedure and its innovations would significantly improve clarity and accessibility.

We have now included a high-level flow chart in the revised manuscript (Figure 1). It includes all the network recovery steps we have presented in the manuscript and shows all the available options for the reader. This makes the central points of the manuscript easier to grasp.

Comment 7: The manuscript should more clearly explain the differences between the generalized expectation-maximization (GEM) algorithm and the standard EM algorithm, and elaborate on why GEM is particularly effective in the context of this model.

We have now expanded the explanation of the GEM algorithm to further improve the readability of the manuscript. For our model, the EM algorithm is not possible to implement because the M-step (maximization step) has to be calculated in parts, in order to have closed-form solutions. Our GEM algorithm can be referred to as the ECM (expectation-conditional-maximization) algorithm or the conditional modes algorithm. However, we decided to adopt the name GEM because it is an umbrella term and includes different versions of the EM and ECM algorithms.

We expanded the GEM explanation to further improve the readability of the manuscript.

Comment 8: The performance comparisons across methods rely on fixed α and CI strategies for the proposed model. To ensure fairness, I suggest applying a consistent criterion (e.g., a fixed FDR threshold such as 0.2) across all methods.

In fairness to all methods, it would be a good thing to use a consistent criterion. This makes the comparison easier and fairer. The problem is that not all methods and implementations have a "built-in" FDR control. For tested methods, only our HMFGraph and CLEVEL from the R package `PCGII` have an implemented FDR control methodology.

For other methods, the only possible way to control FDR is to manually adjust the method's tuning parameter so that it results in the desired target FDR level. However, this is an extremely exhaustive task to perform, and on the other hand, it is only possible for simulated datasets, as they have ground truth. Therefore, the FDR-controlled results would not reflect the methods' real performance.

We have now included a more thorough explanation about the FDR control, and that only HMFGraph and CLEVEL have the FDR control procedure in the Simulated scale-free network datasets section.

Comment 9: Although real biological datasets are analyzed, the interpretations remain largely qualitative. Incorporating more biological validation—such as comparison with known gene interactions or pathway annotations—would greatly strengthen the biological significance of the recovered networks.

We appreciate the suggestion that the real data results need more interpretation. We have now added larger explanation sections on both real example datasets.

For the riboflavin results, we change the investigation such that the riboflavin production is its own node in the recovered network. Now we can more broadly find comparisons to our results and investigate which genes are linked to riboflavin production. In Table 5, we show all genes that are connected to riboflavin production in the network. This means that the expressions of those genes are partially correlated with the riboflavin production and can have some biological interaction. We also found that one cluster that is connected to the riboflavin production has previously been identified, and the genes in it have proven biological interactions. The idea of this example analysis is that GGM can show complex variable connections and correlation with an easy-to-interpret form.

For the American gut dataset, we included discussion and interpretation of the clusters found. From our results, we noticed that OTUs from different taxonomic groups have less significant partial correlations than those from the same taxonomic group. This has been observed with the same dataset and also with other gut datasets using other GGM methods. Our results suggest that OTUs from taxonomic groups are independent of each other, and OTUs from the same group often can be found with each other. This behavior can be seen with taxonomic levels *phylum*, *order*, and *family*.

The results with the expanded interpretation section are now in the revised manuscript in sections Riboflavin dataset and American gut dataset.

Comment 10: Given the large number of mathematical symbols (e.g., α, β, ν, δ), I recommend including a notation table in the methods section to improve readability and reduce potential confusion.

We appreciate this suggestion and believe that including a notation table can indeed enhance readability. We have now added a notation table in the revised manuscript (Table 1). The table has descriptions of all important mathematical symbols so that the manuscript and the method are more approachable.

Reviewer 2

Comment 1: The introduction section of this paper is too statistically technical. As this paper is motivated by constructing biological networks, it would be more interesting to general audiences if the authors can make clear which kind of biological network you want to recover, protein network, gene network, or gut network, and introduce some neccessary biological backgrounds.

In the revised manuscript, we added a new paragraph to the introduction section that gives background for biological networks. We also included more discussion on features common in biological data and how our method is well-suited for them.

Comment 2: My second question is about the validity of the Gaussian assumption. Many biological data do not fully follow the Gaussian assumption and also exhibit heterogeneity, such as the single cell gene expressions. In the real applications, the paper analyzes gene expression data and gut data. Therefore, it is important to verify the Gaussian assumption in this data. In my opinion, one important statistical paper to address single cell gene expression data is "Estimating heterogeneous gene regulatory networks from zero-inflated single-cell expression data", The Annals of Applied Statistics, 2022. The authors may emphasize the difference between the proposed method HMFGraph and this AOAS paper, and I believe this AOAS paper can further motivate the improvement of HMFGraph when applied to zero-inflated and heterogeneous biological data.

In the revised manuscript, we added more discussion about the Gaussian assumption, data normalization and complications of real biological datasets (in the Discussion section). In particular, it is true that with some biological datasets, the Gaussian assumption is not correct. This is important to take into account because it can change the results significantly. There are different approaches to handle this problem, for example, normalization (transforming the data such that the Gaussian assumption is more appropriate) or taking the unique data structure into account with the model, as was done in [1]. Their approach is impressive and seems to work well with their use case.

We followed the approach of using a nonparanormal transformation to ease with the Gaussian assumption [2]. This was used with both real example datasets. The benefit of the nonparanormal transformation is that it can be

used with all data types. Based on our empirical experiments, the nonparanormal transformation helps in settings where the data is not normally distributed, and it is a useful tool for GGMs.

Comment 3: Regarding the edge selection in section 2.5, why do not the authors apply a variable selection prior to the edges, such spike-slab prior or horseshoe prior? The authors should provide some explanations.

The edge selection step is required with the Matrix-F prior. This is because the prior induces a uniform regularization to all off-diagonal elements, but will not shrink them exactly to zero. More generally, methods like spike-and-slab or horseshoe prior would produce a truly sparse precision matrix estimate and could thus omit an edge selection step. However, often with these methods, an additional edge selection is still included to improve control of false positives, or even required to achieve a sparse precision matrix estimate. For example, in [3], a graphical horseshoe was used for network estimation, but the method still required a post variable/edge selection step by using credible intervals of the estimated precision matrix. With spike-and-slab prior, [4] and [5] selected the edges/variables by thresholding posterior inclusion probabilities.

We have now clarified these points above in the revised manuscript in Edge selection with credible intervals section.

Comment 4: About the hyperparameter selection, users should calculate Cond($\hat{\Omega}_\alpha$) for each alpha. What is the computational time for one single alpha? Is it practically applicable? Moreover, could the authors implement some sensitivity analyses for these hyperparameters? In this way, we can largely reduce the selection range of the hyperparameters.

For a single α, the proposed GEM algorithm has to be run once. By our experimentation, the α selection takes approximately 5-10 rounds of the GEM algorithm, i.e., the GEM has to be run 5-10 times. This will increase the computation times noticeably. For instance, with $p = 400$, a single run of the GEM will take about 14 s (Table 1) and the α selection could then take approximately 140 s.

Parallelization cannot be utilized for the binary search, but in order to improve computation times, we use the so-called ”warm start” trick [6, 7]. This means that for the next GEM algorithm run with α_{new}, the previous results (with α_{old}) $\widehat{\Omega}_{\alpha_{old}}$ are used as its initial values. If α_{old} and α_{new} are close, the improvement for the convergence is noticeable. The rows HMFGraph-P-α^3 (with permutations using parallelization) and HMFGraph-P-α^4 (with permutations without using parallelization) include the α selection calculations with the

"warm start" trick. The calculations still take less time than MCMC samplers (with 5000 iterations).

We noticed that if α is close to 1, the algorithm takes longer to converge. Also, results with $\alpha = 0.95$ vs results with $\alpha = 0.99$ are in practice the same, but with $\alpha = 0.99$ the convergence is noticeably slower. For this reason, α_{max} is always set to 0.95 for all results shown. In addition, the lower limit for α was always set to $p/(p+n)$. This makes the algorithm slightly faster, and this lower limit is not a problem for any of our test datasets.

In the revised manuscript, we increased the explanation of the condition number constraint method (CC-method), and also included a more detailed explanation of the binary search algorithm in the Supporting Information.

Comment 5: The explanations for the results in real datasets are shallow. Could the authors provide more biological insights from the results by HMFGraph? More biological findings rather than simulation results are more interesting to Plos computational biology readers.

We appreciate the suggestion that the real data results need more interpretation. We have now added larger explanation sections on both real example datasets.

For the riboflavin results, we change the investigation such that the riboflavin production is its own node in the recovered network. Now we can more broadly find comparisons to our results and investigate which genes are linked to riboflavin production. In Table 5, we show all genes that are connected to riboflavin production in the network. This means that the expressions of those genes are partially correlated with the riboflavin production and can have some biological interaction. We also found that one cluster that is connected to the riboflavin production has previously been identified, and the genes in it have proven biological interactions. The idea of this example analysis is that GGM can show complex variable connections and correlation with an easy-to-interpret form.

For the American gut dataset, we included discussion and interpretation of the clusters found. From our results, we noticed that OTUs from different taxonomic groups have less significant partial correlations than those from the same taxonomic group. This has been observed with the same dataset and also with other gut datasets using other GGM methods. Our results suggest that OTUs from taxonomic groups are independent of each other, and OTUs from the same group often can be found with each other. This behavior can be seen with taxonomic levels *phylum*, *order*, and *family*.

The results with the expanded interpretation section are now in the revised manuscript in sections Riboflavin dataset and American gut dataset.

Reviewer 3

Comment 1: Justification of the introduced new parameter β: Figure 3 showed the curves of FDR with varying α, however, the role and selection process for β require further explanation or empirical validation to ensure readers understand its impact on network recovery.

To address this, we have now included three new figures that show how the β values change the precision matrix estimation and the recovered network. In the first new figure (Figure 2), we showcase how increasing β values will decrease the values of the off-diagonal elements. This will, in turn, decrease the number of connections found in the network (see Supplementary Figure S7). The impact of β-value is less noticeable if α is selected using the CC-method (see Supplementary Figures S6 and S7).

Comment 2: Sensitivity/Specificity Analysis: The authors reported the FDR of varying α. Reporting sensitivity/specificity for both α and β would help guide users in parameter selection.

Thank you for the suggestion. We have now added similar FDR control plots with varying β values. These plots are in the supporting information file (Figures S8 and S9). Based on these and figures with varying α-values, it can be noted that α-values have a greater impact on network recovery accuracy than β-values.

Comment 3: Biological Interpretation: The biological significance of the results, particularly in the gut microbiome study, could be strengthened with a comparison to results in the literature.

We appreciate the suggestion that the real data results need more interpretation. We have now added larger explanation sections on both real example datasets.

For the riboflavin results, we change the investigation such that the riboflavin production is its own node in the recovered network. Now we can more broadly find comparisons to our results and investigate which genes are linked to riboflavin production. In Table 5, we show all genes that are connected to riboflavin production in the network. This means that the expressions of those genes are partially correlated with the riboflavin production and can have some biological interaction. We also found that one cluster that is connected to the riboflavin production has previously been identified, and the genes in it have proven biological interactions. The idea of this example analysis is that GGM can show complex variable connections and correlation with an easy-to-interpret form.

For the American gut dataset, we included discussion and interpretation of the clusters found. From our results, we noticed that OTUs from different taxonomic groups have less significant partial correlations than those from the same taxonomic group. This has been observed with the same dataset and also with other gut datasets using other GGM methods. Our results suggest that OTUs from taxonomic groups are independent of each other, and OTUs from the same group often can be found with each other. This behavior can be seen with taxonomic levels *phylum*, *order*, and *family*.

The results with the expanded interpretation section are now in the revised manuscript in sections Riboflavin dataset and American gut dataset.

Comment 4: page 5/23, line 135, $\beta \in [0, 1[$ should be changed to $\beta \in [0, 1]$

Thank you for noticing this mistake. The parameter β can obtain the value 1 if and only if $\nu = p + 1$:

$$\beta = \frac{\delta + p - 1}{\delta + \nu - 2} = \frac{\delta + p - 1}{\delta + p - 1} = 1. \tag{1}$$

This error is now corrected in the revised manuscript.

References

[1] Q. Wu and X. Luo, "Estimating heterogeneous gene regulatory networks from zero-inflated single-cell expression data," *The Annals of Applied Statistics*, vol. 16, no. 4, pp. 2183–2200, 2022.

[2] H. Liu, J. Lafferty, and L. Wasserman, "The nonparanormal: Semiparametric estimation of high dimensional undirected graphs," *Journal of Machine Learning Research*, vol. 10, no. 80, pp. 2295–2328, 2009.

[3] Y. Li, B. A. Craig, and A. Bhadra, "The graphical horseshoe estimator for inverse covariance matrices," *Journal of Computational and Graphical Statistics*, vol. 28, no. 3, pp. 747–757, 2019.

[4] M. Byrd, L. H. Nghiem, and M. McGee, "Bayesian regularization of gaussian graphical models with measurement error," *Computational Statistics & Data Analysis*, vol. 156, p. 107085, 2021.

[5] L. Gan, X. Yang, N. Narisetty, and F. Liang, "Bayesian joint estimation of multiple graphical models," *Advances in Neural Information Processing Systems*, vol. 32, 2019.

[6] X. Li, T. Zhao, X. Yuan, and H. Liu, "The flare package for high dimensional linear regression and precision matrix estimation in R," *Journal of Machine Learning Research*, 2015.

[7] J. Kim, S. Kim, and S. Choi, “Learning to warm-start Bayesian hyperparameter optimization,” 2018.
