# Supplementary material for: HMFGraph: Novel Bayesian approach for recovering biological networks
Source: PLoS Comput Biol. 2025 Oct 30;21(10):e1013614. doi: 10.1371/journal.pcbi.1013614 (PMC12591435; doi:10.1371/journal.pcbi.1013614)
Supplement: Attachment — Submitted filename: Response_to_Reviewers_auresp_2.pdf [file pcbi.1013614.s003.pdf]

Detailed answers to reviewers' comments. PCOMPBIOL-D-25-00977 HMFGraph: Novel Bayesian approach for recovering biological networks PLOS Computational Biology Revision 2

Korhonen et al.

October 2025

Reviewer 1

Comment 1: The flowchart is rather simplistic. Please enhance it with more relevant illustrations for better visualization and also further optimize the layout and spacing of the figures.

Thank you for the comment. We have now enhanced the flowchart by including visualizations of precision and adjacency matrices, and network structure. This improved the flowchart and made it visually more interesting. In addition, the layout is optimized. The new flowchart is now presented in the revised manuscript as Figure 1. We believe that the new flowchart is more informative and helps readers to follow the main points of the article.

Comment 2: In light of the authors' contributions to biological networks, I suggest that the authors consider citing the following article at appropriate places in the manuscript. DOI:10.1038/s41551-024-01312-5 DOI: 10.1109/JBHI.2025.3585290

Thank you for these suggestions. Both articles were interesting and relevant for network analysis. The first suggestion [1] was more closely related to our

work and now has been cited in the Introduction section in the second paragraph. This will enrich the Introduction section by giving more context on how recovered biological networks can be further analyzed.

References

[1] X. Su, P. Hu, D. Li, B. Zhao, Z. Niu, T. Herget, P. S. Yu, and L. Hu, "Interpretable identification of cancer genes across biological networks via transformer-powered graph representation learning," *Nature Biomedical Engineering*, vol. 9, no. 3, pp. 371–389, 2025.
